# Circular RNA circFIRRE drives osteosarcoma progression and metastasis through tumorigenic-angiogenic coupling

**DOI:** 10.1186/s12943-022-01624-7

**Published:** 2022-08-19

**Authors:** Lingfeng Yu, Hao Zhu, Zhen Wang, Jianhao Huang, Yan Zhu, Gentao Fan, Yicun Wang, Xi Chen, Guangxin Zhou

**Affiliations:** 1 Department of Orthopedics, Affiliated Jinling Hospital, Medical School of Nanjing University, Nanjing, 210002 Jiangsu China; 2 Department of Orthopedics, Affiliated Jianhu Hospital of Nantong University, Jianhu, Yancheng, 224700 Jiangsu China; 3 Nanjing Medical University, Nanjing, 210029 Jiangsu China; 4 Nanjing Drum Tower Hospital Center of Molecular Diagnostic and Therapy, State Key Laboratory of Pharmaceutical Biotechnology, Jiangsu Engineering Research Center for MicroRNA Biology and Biotechnology, School of Life Sciences, Nanjing University, Nanjing, 210023 Jiangsu China

**Keywords:** Osteosarcoma, Angiogenesis, circFIRRE, YY1, miR-486-3p, miR-1225-5p, LUZP1

## Abstract

**Background:**

Disappointing clinical efficacy of standard treatment has been proven in refractory metastatic osteosarcoma, and the emerging anti-angiogenic regimens are still in the infantile stage. Thus, there is an urgent need to develop novel therapeutic approach for osteosarcoma lung metastasis.

**Methods:**

circFIRRE was selected from RNA-sequencing of 4 matched osteosarcoma and adjacent samples. The expression of circFIRRE was verified in clinical osteosarcoma samples and cell lines via quantitative real-time polymerase chain reaction (RT-qPCR). The effect of circFIRRE was investigated in cell lines in vitro models, ex vivo models and in vivo xenograft tumor models, including proliferation, invasion, migration, metastasis and angiogenesis. Signaling regulatory mechanism was evaluated by RT-qPCR, Western blot, RNA pull-down and dual-luciferase reporter assays.

**Results:**

In this article, a novel circular RNA, circFIRRE (hsa_circ_0001944) was screened out and identified from RNA-sequencing, and was upregulated in both osteosarcoma cell lines and tissues. Clinically, aberrantly upregulated circFIRRE portended higher metastatic risk and worse prognosis in osteosarcoma patients. Functionally, in vitro, ex vivo and in vivo experiments demonstrated that circFIRRE could drive primary osteosarcoma progression and lung metastasis by inducing both tumor cells and blood vessels, we call as “tumorigenic-angiogenic coupling”. Mechanistically, upregulated circFIRRE was induced by transcription factor YY1, and partially boosted the mRNA and protein level of LUZP1 by sponging miR-486-3p and miR-1225-5p.

**Conclusions:**

We identified circFIRRE as a master regulator in the tumorigenesis and angiogenesis of osteosarcoma, which could be purposed as a novel prognostic biomarker and therapeutic target for refractory osteosarcoma.

**Supplementary Information:**

The online version contains supplementary material available at 10.1186/s12943-022-01624-7.

## Introduction

Osteosarcoma (OS) is the most common malignant primary bone tumor most often occurring in children and adolescent during their pubertal growth spurts [1, 2]. The most common primary site of OS is the metaphysis of the long diaphysis, especially in the proximal tibia and distal femur. The main metastasis route of OS is the lung, which greatly reduces the 5-year survival rate from 70% in non-metastatic patients to 20% [3, 4]. Angiogenesis induced during tumorigenesis and distant recurrence can serve as a valuable parameter for predicting the outcome in OS patients [5, 6]. As one of the integral hallmarks of cancer, angiogenesis can supply oxygen and nutrients, as well evacuate carbon dioxide and metabolites [7]. Despite exciting achievements reported in some pilot studies [5, 6, 8, 9], the exploration of anti-angiogenesis in refractory OS remains in its infantile stage.

Circular RNAs (circRNAs) are one of the subclasses of RNA molecules that stem from corresponding genes consisting of exons, introns or intergenic spacers. Unlike linear RNAs, circRNAs are produced by back-splicing with the 3′ and 5′ splice-donor site concatenated covalently, thus resistant to exonuclease degradation and highly stable [10, 11]. CircRNA dysregulation is reportedly involved in various diseases [12–14]. Mechanistically, circRNAs possess miRNA-binding domains and commonly exert regulatory effects by sponging certain miRNAs to post-transcriptionally modulate gene level [10]. The role of circRNAs in OS has been reported in many studies [15–18], but the exact action mechanism remains unclear.

In this study, we detected the aberrantly expressed circRNAs between 4 paired OS and histologically normal adjacent samples by RNA-seq assay, and then further identified and characterized a circRNA comprised of exon 5 to 10 of the Firre Intergenic Repeating RNA Element (FIRRE) gene, which was termed as circFIRRE (hsa_circ_0001944). Finally, we explored the function and underlying mechanism of circFIRRE in the growth, metastasis and angiogenesis of OS.

## Patients and Methods

### Patients and samples

A total of 114 surgically resected freshly frozen OS samples and their corresponding adjacent samples (cohort 1, 2, 3, 4) were obtained from OS patients at Affiliated Jinling Hospital, Medical School of Nanjing University (Nanjing, China). The histopathological diagnosis of the resected samples was confirmed based on the criteria from World Health Organization (WHO). Among these samples, 4 fresh paired samples (cohort 1) were obtained and applied for RNA-sequence in March 2016, and the remaining 104 paired samples (cohort 2, 3) obtained from January 2008 to June 2020 were used for expression validation of circRNA in this study. For circRNAs screening, 16 paired samples obtained from January 2015 to February 2016 (cohort 2) were employed. For detecting the expression level of transcription factor YY1, miRNAs, and LUZP1, 35 paired samples obtained from April 2016 to June 2020 (cohort 3) were employed. For magnetic bead cell sorting, 6 paired samples obtained in June 2022 (cohort 4) were employed. All samples were preserved in liquid nitrogen for long-term storage. Informed consent was signed by participating patients or their legally authorized representatives to utilize their clinical data and surgical samples, and the study protocol was ratified by the Ethics Committee of the said hospital (2016NZKY-020-03). The detailed clinical baseline data of 104 paired samples (cohort 2, 3) are described in Fig. S2A.

### Statistical analysis

All experiments were performed in triplicate, and representative experimental data are presented as the mean value ± standard deviation (SD). Two group comparisons were calculated by the t tests; unidimensional multiple groups comparisons were calculated by one-way ANOVA tests; bidimensional multiple group comparisons were calculated by two-way ANOVA tests using GraphPad Prism 9.20 (GraphPad, Inc., LaJolla, CA). Statistical analysis and visualization of clinical data and RNA-sequencing data were implemented in R (version: 3.6.3). The R packages that we used are as follows: volcano plots, GSEA, correlation analysis plots, forest plots and risk score plots: ggplot2 package; heatmap: ComplexHeatmap package; GSEA statistical analysis: clusterProfiler package [19]; GSEA annotation database, MSigDB Collections(https://www.gsea-msigdb.org/gsea/msigdb/index.jsp) ; Kaplan-Meier analyses: survminer package and survival package. *P < 0.05; **P < 0.01; ***P < 0.001; ****P < 0.0001; ns = not significant.

The detailed experimental procedures are described in the Additional file 1.

## Results

### Screening and characterization of circFIRRE in OS samples and cells

**Fig. 1:**
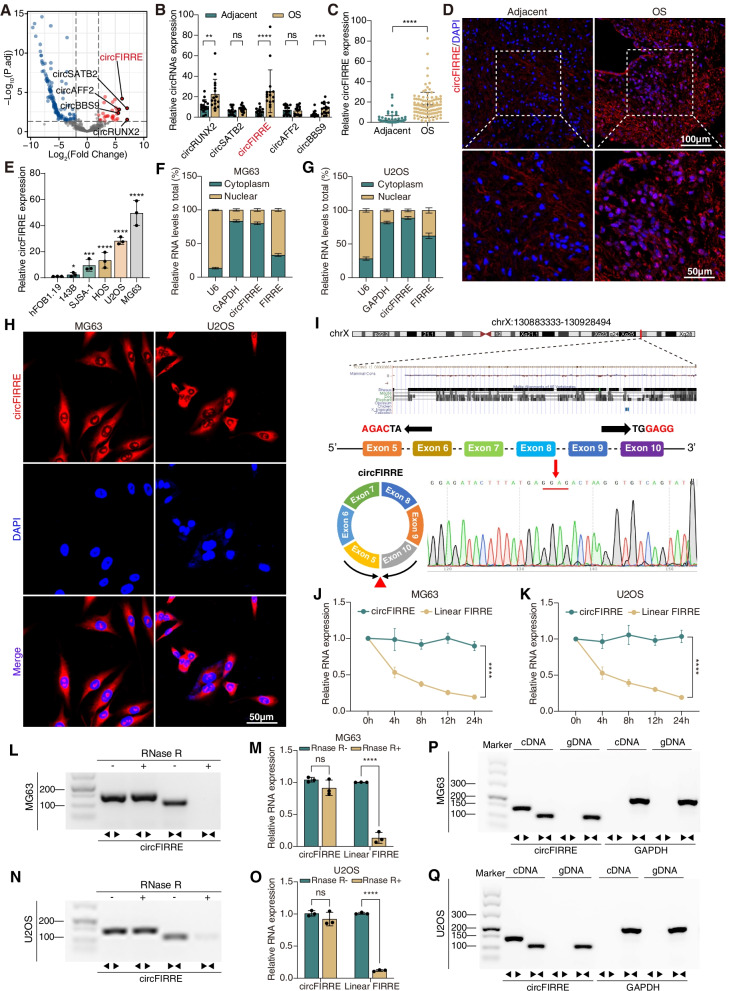
Identification and validation of upregulated circFIRRE in OS tissues and cells. **A** A Volcano plot illustrating the differential expression of circRNA in RNA-sequencing, where the red and blue points represent upregulated and downregulated circRNAs that met screening criteria as described. **B** Validation of differentially expressed circular RNAs by RT-qPCR (*n*=3 in each group). **C** The relative expression of circFIRRE in 104 paired clinical OS samples and normal controls were analyzed by RT-qPCR. **D** RNA–fluorescence in situ hybridization assays (FISH) were conducted in clinical OS and normal tissues to demonstrate circFIRRE expression using Cy3-labeled probes (red); DAPI-stained nuclei (blue). Scale bars=100 μm and 50 μm. **E** The relative expression of circFIRRE in human OS cell lines and normal osteoblasts (*n*=3 in each group). **F**-**G** RT-qPCR indicated the abundance of circFIRRE in cytoplasm of MG63 and U2OS cells. GAPDH and U6 were used as positive controls for cytoplasm and nucleus (*n*=3 in each group). **H** FISH assay demonstrated that circFIRRE was predominantly localized in the cytoplasm of MG63 and U2OS cells. Red indicates circFIRRE; DAPI-stained nuclei (blue). Scale bar=50 μm. **I** Schematic illustration presents the formation of circFIRRE. The trans-splicing site of circFIRRE validated by Sanger sequencing is highlighted by the red underline. **J**-**K** The relative expression of circFIRRE and linear FIRRE was analyzed by RT-qPCR at different time points after Actinomycin D treatment in MG63 and U2OS cells (*n*=3 at each time point). **L**-**O** The relative expression of circFIRRE and linear FIRRE was detected by RT-PCR and RT-qPCR in MG63 and U2OS cells in the presence of RNase R. **P**-**Q** The presence of circFIRRE was validated in MG63 and U2OS cells by RT-PCR. circFIRRE was amplified by divergent primers in cDNA, but not in genomic DNA. Values are presented as mean ± SD; the bar charts, line charts, error bars and dots represent the quantitative analysis of 3 independent experiments; (**B**, **C**, 2-tailed Student t test; **E**, one-way ANOVA; **J**, **K**, **M** and **O**, two-way ANOVA); *P < 0.05; **P < 0.01; ***P < 0.001; ****P < 0.0001; ns = not significant

Initially, we studied the circRNAs expression profile using ribosomal RNA-depleted RNA-seq analysis from 4 paired OS and control samples. In the circRNAs profile database constructed after sequencing, most sequence-length of circRNAs were less than 2000 nucleotides (Fig. S1A). Aberrantly expressed circRNAs were presented in Volcano Plot (Fig. 1A). Normalization and differential expression analysis of circRNAs was performed using DESeq2 package. The screening thresholds for differentially expressed circRNAs (DECs) was ∣Log_2_(Fold change) ∣ >2, adjusted *P* < 0.05, and basemean value >3, and the DECs were depicted in volcano plots (Fig. 1A). Among 163 DECs, 30 circRNAs were upregulated and presented in heatmap (Table S1, Fig. S1B). The top 5 upregulated circRNAs that sorted by Log_2_(Fold change) were included in further screening, containing circRUNX2 (hsa_circ_0003563), circSATB2 (hsa_circ_0003915), circFIRRE (hsa_circ_0001944), circAFF2 (hsa_circ_0001947) and circBBS9 (hsa_circ_0003162). Then, we expanded the OS sample size to 16 pairs and detected expression level of the 5 DECs using RT-qPCR analysis. As shown in Fig. 1B, circFIRRE (Circbase [20] ID: hsa_circ_0001944) was identified the most significant DEC for follow up study since the biggest difference between OS tissues and adjacent tissues. Under further verification in a bigger sample cohort, circFIRRE was significantly upregulated in 104 matched clinical OS samples and adjacent normal samples using RT-qPCR analysis (Fig. 1C). The upregulated tendency was also confirmed by FISH assay in 5 paired fresh patient samples (Fig. 1D, Fig. S1C). Meanwhile, the expression level of circFIRRE was significantly elevated in 5 OS cell lines compared to osteoblast cell line (hFOB1.19), especially in MG63 and U2OS (Fig. 1E).

Subcellular localization of circFIRRE is essential for its cellular function, lncLocator 2.0 [21] was firstly applied to predict circFIRRE subcellular localization, and the result showed that circFIRRE was preponderantly distributed in the cytoplasm (Fig. S1D). In addition, robust expression of cytoplasmic circFIRRE in MG63 and U2OS (about 80%) was confirmed by both RT-qPCR following nuclear/cytosol separation (Fig. 1F-G) and RNA FISH (Figure H, Fig. S1E), while only a small fraction of circFIRRE (about 20%) were localized in the nucleus. circFIRRE is originated from firre intergenic repeating RNA element (FIRRE) locus on chromosome X (chrX: 130883333 - 130928494), and generated by the exon 5 to 10 of gene FIRRE region. The exact size of circFIRRE is 1096 bp and its specific trans-splicing was confirmed by Sanger sequencing (Fig. 1I). The stability of circFIRRE in MG63 and U2OS cells was examined after treatment with actinomycin D, knowing that it can transcriptionally inhibit RNA synthesis. circFIRRE had a half-life of >24 hours, which was more stable than linear transcript FIRRE with a half-life of <5 hours (Fig. 1J-K). circFIRRE was also resistant to digestion with exonuclease ribonuclease R (RNase R), while FIRRE was readily degraded after treatment (Fig. 1L-O). These results suggested that OS cells could sustainedly and stably express circFIRRE, which may be an eligible biomarker for diagnosis or prognosis prediction.

Knowing that back-spliced junction could come from either trans-splicing or genomic rearrangements, agarose gel electrophoresis (AGE) was applied to eliminate the possibility of genomic rearrangements. circFIRRE and liner FIRRE were separately amplified by divergent primers and convergent primers in cDNA and genomic DNA extractives from OS cells. circFIRRE was only found in cDNA but not in genomic DNA by AGE assay (Fig. 1P-Q).

### Upregulated circFIRRE expression portends higher metastatic risk and worse prognosis in OS patients

**Fig. 2:**
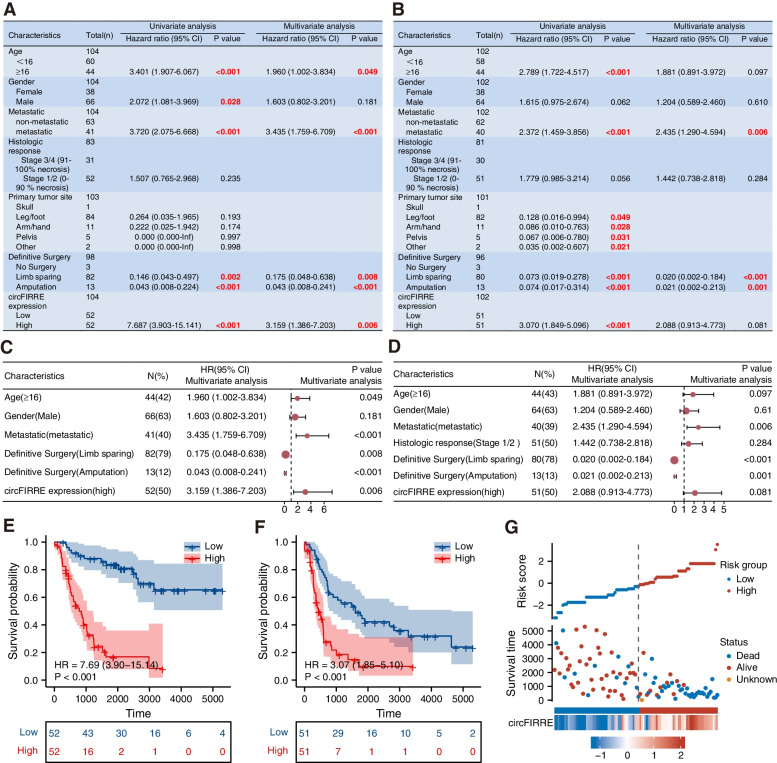
Identification of circFIRRE as an independent risk factor for predicting OS prognosis. **A**-**B** Risk factors related to overall survival (OS) and disease-free survival (DFS) were identified by univariate and multivariate Cox regression analysis, and risk factors with significant difference are highlighted in red. **C**-**D** Forest plots of univariate and multivariate analysis for risk factors associated with OS and DFS. **E** Kaplan–Meier curves for OS, high circFIREE expression versus low circFIREE expression. **F** Kaplan–Meier curves for DFS, high circFIREE expression versus low circFIREE expression. **G** Multidimensional analysis of association between the circFIRRE expression level, clinical outcome (survival time, status) of osteosarcoma patients and risk score

According to the circFIRRE expression in OS lesions, the 104 patients were equally divided into two groups. The baseline characteristics of the enrolled patients are displayed in Fig. S2A. To evaluate risk factors in overall survival (OS) and disease-free survival (DFS), univariate and multivariate analyses were applied. Univariate analysis showed that age, surgical modalities, tumor metastasis and high circFIRRE expression were associated with OS and DFS. In compromising these variables, multivariate analysis illustrated that metastasis and elevated circFIRRE expression were independent risk factors for prognosis of OS patients, together with age and surgical modalities (Fig. 2A-D). Additionally, Kaplan-Meier survival curves showed worse OS and DFS in patients in high circFIRRE expression than those with low circFIRRE expression group (*p*<0.001) (Fig. 2E-F). Specifically, high expression level of circFIRRE was positively associated with shorter survival, poorer clinical outcomes and higher risk scores (Fig. 2G). In addition, clinical baseline demonstrated that a high expression level of circFIRRE was also correlated with a high metastatic ratio (Fig. S2A).

All these results showed that circFIRRE could function as an independent risk factor of OS patients and may participate in OS progression and metastasis.

### circFIRRE promotes OS progression in vitro

To investigate the signature of biological states in OS, we performed gene-set enrichment analysis (GSEA) of hallmark gene sets. As the high-scoring gene sets shown in Fig. S2B-F, angiogenesis, epithelial mesenchymal transition (EMT), G2M check point, E2F targets and mitotic spindle related genes were highly enriched in OS tissues compared to adjacent normal tissues. Therefore, proliferation, migration and angiogenesis in OS got a critical focus in follow-up study.

To explore the impact of circFIRRE upregulation on tumor progression, loss-of-function assays were performed in OS cell lines. In the first step, we designed three siRNAs targeting the back-spliced junction of circFIRRE and conducted transfection in MG63 and U2OS cells. The results showed that si-circFIRRE-1 (si-1) and si-circFIRRE-2 (si-2) had relative a better knockdown effect (Fig. S3A). Afterwards, si-1 and si-2 were chosen for the preliminary experiment in MG63 cells. CCK8 and wound healing assays were performed to test OS cell proliferation and migration capacities. CCK8 assays showed that both si-1 and si-2 significantly diminished the growth rate of MG63, which was consistent with the knockdown efficiency in transfection (Fig. S3B). Wound healing assays showed that si-1 and si-2 retarded the rehabilitation of scratches 24 hours after scratching (Fig. S3C). These results indicate that circFIRRE may contribute to OS progression in vitro.

**Fig. 3:**
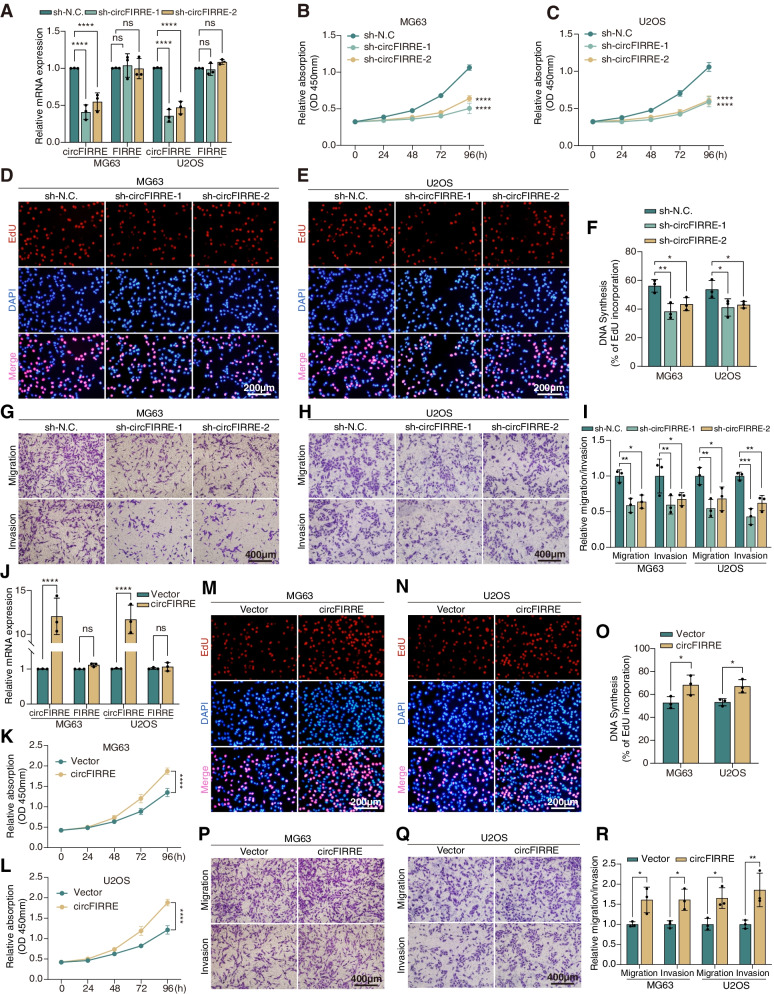
circFIRRE affects OS proliferation, migration and invasion in vitro. **A** Relative mRNA expression of circFIRRE and linear FIRRE in both MG63 and U2OS cells was examined by RT-qPCR after stable infection of either shRNAs lentivirus (sh-circFIRRE-1 and -2) or scramble shRNA lentivirus (sh-N.C.) (*n*=3 in each group). **B**-**C** CCK8 assay was applied to measure the cell viability influenced by circFIRRE knockdown at different time points in both MG63 and U2OS (*n*=6 at each time point). **D**-**F** EdU assay was performed to detect cell proliferation after circFIRRE silencing in MG63 and U2OS. S-phase entry was visualized by EdU incorporation (red); DAPI-stained nuclei (blue). Scar bar=200 μm. Image quantification conducted as described in methods (*n*=5 in each group). **G**-**I** Cell migration and invasion were detected by transwell assay after circFIRRE silencing in MG63 and U2OS cells. Scar bar=400 μm. Image quantification conducted as described in methods (*n*=5 in each group). **J** Relative expression of circFIRRE and linear FIRRE in both MG63 and U2OS cells was detected by RT-qPCR after transfection of circFIRRE overexpression vectors or scramble vectors (*n*=3 in each group). **K**-**L** CCK8 assay was applied to measure the cell viability after circFIRRE overexpression at different time points in MG63 and U2OS (*n*=6 at each time point). **M**-**R** EdU and Transwell assays were employed to detect cell proliferation, migration and invasion after circFIRRE overexpression in MG63 and U2OS. Scar bars=200 μl and 400 μm. Values are presented as mean ± SD; the bar charts, line charts, error bars and dots represent the quantitative analysis of 3 independent experiments; two-way ANOVA was used; *P < 0.05; **P < 0.01; ***P < 0.001; ****P < 0.0001; ns = not significant

In the second step, we infected lentivirus packaged shRNAs (sh-circFIRRE-1 and -2) (Fig. S3D) and established circFIRRE stable knockdown cell lines in MG63 and U2OS (Fig. 3A). Then, we performed CCK8 and EdU assays to evaluate the proliferation ability of OS cells. The result showed that sh-circFIRRE cell proliferation and EdU combination were reduced significantly as compared with the negative control cells (Fig. 3B-F). Additionally, wound healing assay showed that silencing circFIRRE expression suppressed the migration capability of OS cells (Fig. S3E); both migration and invasion abilities of OS cells were diminished by circFIRRE knockdown in Transwell migration and invasion assay (Fig. 3G-I). We then conducted flow cytometry to evaluate the cell cycle and found that sh-circFIRRE cells were arrested in G0/G1 phase (Fig. S3F-H), demonstrating that circFIRRE knockdown could promote cell cycle arrest. Collectively, these results demonstrate that circFIRRE knockdown inhibited OS tumor growth and motility in vitro.

In the third step, gain-of-function assays were performed to further explore the function of circFIRRE in vitro. Firstly, pGMLV-circRNA vector was designed to overexpress circFIRRE in OS cells (Fig. S4A-B) and RT-qPCR verified the overexpression efficiency and confirmed that transfection did not influence linear FIRRE expression (Fig. 3J). Next, CCK8 and EdU assays revealed that circFIRRE overexpression significantly facilitated MG63 and U2OS proliferation (Fig. 3K-O). Additionally, wound healing assay (Fig. S4C) and Transwell assays illustrated that circFIRRE overexpression promoted OS cell migration and invasion (Fig. 3P-R). Flow cytometry demonstrated that circFIRRE promoted cell proliferation by promoting cell cycle (Fig. S4D-F). In summary, circFIRRE contributed to the growth, migration and invasion of OS in vitro.

### circFIRRE can induce angiogenesis

**Fig. 4:**
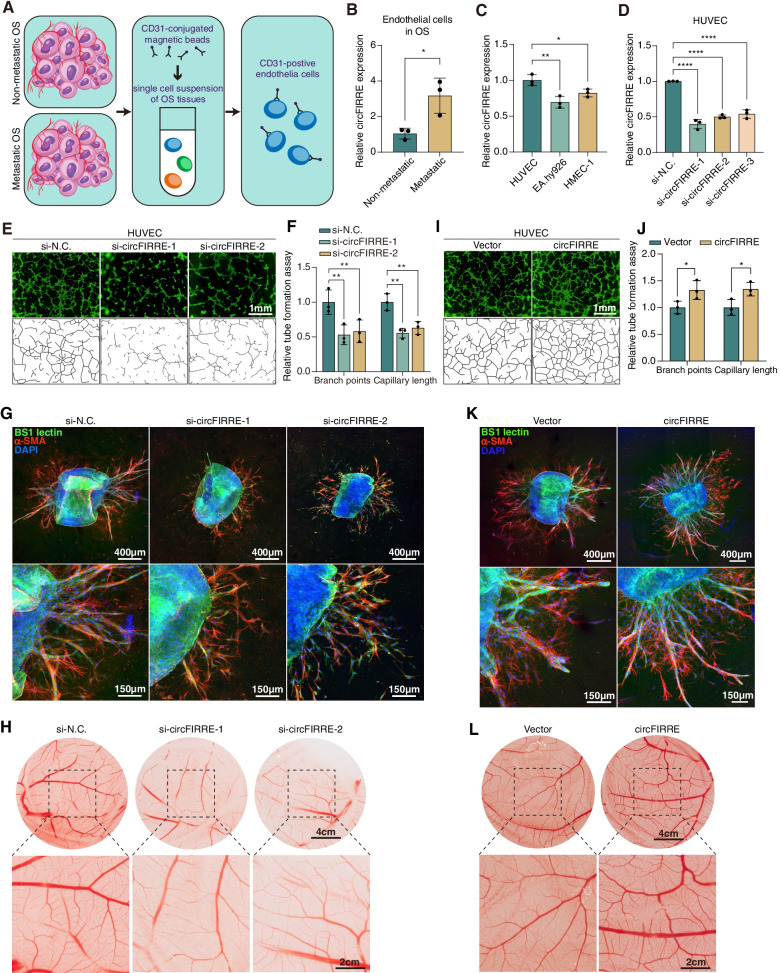
circFIRRE can induce angiogenesis in vitro, ex vivo and in vivo. **A** Schematic description of the flow of magnetic bead cell sorting. **B** Validation of differentially expressed circFIRRE in primary endothelia cells by RT-qPCR (*n*=3 in each group). **C** Validation of differentially expressed circFIRRE in different endothelia cell lines by RT-qPCR (*n*=3 in each group). **D** HUVEC cells were transiently transfected with circFIRRE-siRNAs or scramble siRNA (si-N.C.). 48 hours after transfection, the relative circFIRRE expression in HUVEC cells were detected by RT-qPCR (*n*=3 in each group). **E**-**F** Tube formation assay was applied to determine cell tube formation ability after circFIRRE silencing in HUVEC cells. Scar bar=1mm. Image quantification were conducted as described in methods (*n*=3 in each group). **G** Representative immunofluorescence staining of aortic rings. BS1 lectin-FITC (green) indicated endothelial sprouts; α-SMA-Cy3 (red) stained supporting cells; DAPI-stained nuclei (blue). Scale bars=400 μm and 150 μm. **H** Representative images of CAM photographed on plastic dished after resection from eggs. Scale bars=4 cm and 2 cm. **I**-**L** Tube formation, aortic ring and CAM assays were conducted to examine angiogenesis level induced by circFIRRE overexpression, respectively. Values are presented as mean ± SD; the bar charts, error bars and dots represent the quantitative analysis of 3 independent experiments; (**B**, 2-tailed Student t test; **C**, **D**, one-way ANOVA; **F**, **J**, two-way ANOVA); *P < 0.05; **P < 0.01; ***P < 0.001; ****P < 0.0001

As mentioned before, GSEA analysis found that circFIRRE may contribute the tumor progression and metastasis, and angiogenesis may be the major biological procedure of tumor metastasis in which circFIRRE was involved (Fig. S2B). Hence, we hypothesized that circFIRRE may be involved in angiogenesis, which is vital in OS pulmonary metastasis. The major premise of the pro-angiogenic and pro-metastatic capacities of circFIRRE is that the enrichment of circFIRRE in endothelial cells within the metastatic OS tissues. Therefore, we performed magnetic beads sorting experiment to detect the differential expression of circFIRRE in endothelial cells. Sorted by CD31+ magnetic beads, primary endothelial cells were obtained from both three samples from patients with localized OS and three clinical samples from patients with metastatic OS (Fig. 4A). RT-qPCR analysis demonstrated that circFIRRE was significantly upregulated in endothelial cells within metastatic OS relative to which in endothelial cells within non-metastatic OS (Fig. 4B), revealed its potential function in angiogenesis and OS metastasis.

Among three common endothelial cell lines (HUVEC, EA.hy926, HMEC-1), HUVEC cells exhibited the highest level of circFIRRE expression. Therefore, we chose HUVEC for angiogenesis research to observe the effect of circFIRRE on cell proliferation, migration and tube formation capacities. First, we transferred three circFIRRE targeted siRNAs into HUVEC cells, and found that si-1 and si-2 diminished more than 50% circFIRRE expression level (Fig. 4D), exhibiting a relatively good knockdown effect. Therefore, we selected them for functional experiments. Second, we verified the role of circFIRRE in HUVEC proliferation, motility and tube formation by CCK8 (Fig. S5A), EdU assay (Fig. S5B-C), wound healing assay (Fig. S5D), Transwell migration assay (Fig. S5E-F) and tube formation assay (Fig. 4E-F). It was found that si-circFIRRE significantly compromised cell proliferation, migration and tube formation capabilities in HUVEC cells compared to the control. Third, we used ex vivo aortic ring endothelial cell sprouting assay and in vivo chick chorioallantoic membrane (CAM) assay to further verify our hypothesis. The aortic ring assay showed that si-circFIRRE transfected aortic rings were embedded in collagen for seven days, and less microvessel area was observed as compared with the normal group (Fig. 4G, Fig. S5G-H). CAM is a highly vascularized extraembryonic membrane of the chick embryo, in which newly formed vessels were obviously compromised after si-circFIRRE co-incubation (Fig. 4H, Fig. S5I-J). All these findings demonstrated that circFIRRE knockdown significantly restrained angiogenesis.

Then, we transfected circFIRRE plasmids into HUVEC cells and the overexpression efficiency assessed by RT-qPCR is shown in Fig. S6A. Cell viability and migration capabilities of HUVEC cells were evaluated by CCK8, EdU, wound healing and Transwell migration assays, and the results showed that circFIRRE was vital in maintaining proliferation and migration of HUVEC cells (Fig. S6B-G). Tube formation assay was performed to assess new sprout and the result showed that circFIRRE overexpression prominently increased the branch points and capillary length of HUVEC cells (Fig. 4I-J). CAM and aortic ring assays illustrated that circFIRRE could promote angiogenesis as well (Fig. 4K-L, Fig. S6H-K). Finally, all these experiments revealed that circFIRRE can facilitate neovascularization in neoplasm metastasis.

### The expression of circFIRRE in OS can be regulated by YY1

**Fig. 5:**
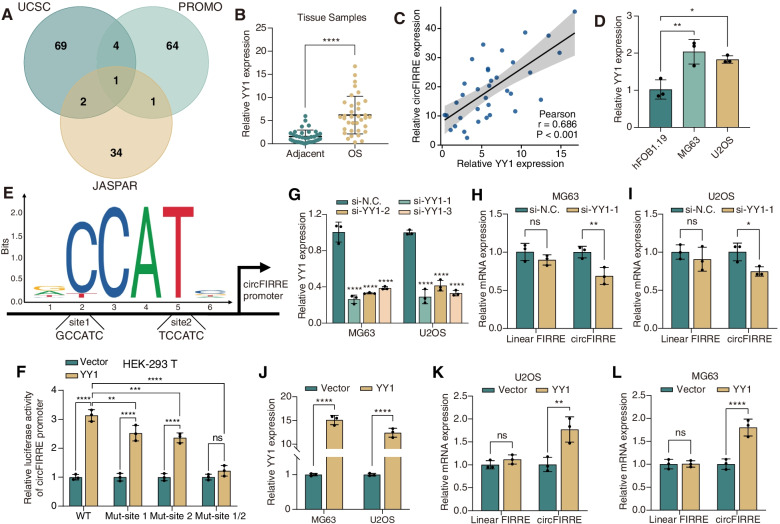
YY1 activates circFIRRE transcription. **A** Wayne diagram showed the overlap between transcription factors of circFIRRE predicted by three different algorithms. **B** Relative mRNA expression of YY1 in 35 paired OS and adjacent normal samples were examined by RT-qPCR. **C** Pearson correlation analysis indicated that YY1 was positively correlated with circFIRRE in 35 paired samples. **D** The relative expression of YY1 was detected in chosen OS cell lines (MG63, U2OS) and control osteoblasts (*n*=3 in each group). **E** Schematic illustration of the binding motif of YY1 and the two possible YY1 binding sites in circFIRRE promoter. **F** Dual luciferase assay was performed to confirm the binding between YY1 and circFIRRE promoter, as well as two specific binding sites in HEK-293 T cells (*n*=3 in each group). **G** Three YY1-targeted siRNAs were transfected into MG63 and U2OS cells, and relative mRNA of YY1 were estimated by RT-qPCR (*n*=3 in each group). **H**-**I** The relative circFIRRE and linear FIRRE expression were detected in MG63 and U2OS cells after YY1 silencing by RT-qPCR (*n*=3 in each group). **J**-**L** The relative YY1, circFIRRE and linear FIRRE expression were detected in MG63 and U2OS cells after YY1 overexpression by RT-qPCR (*n*=3 in each group). Values are presented as mean ± SD; the bar charts, error bars and dots represent the quantitative analysis of 3 independent experiments; (**B**, **F**, 2-tailed Student t test; **D**, one-way ANOVA; **G**-**L**, two-way ANOVA); *P < 0.05; **P < 0.01; ***P < 0.001; ****P < 0.0001; ns = not significant

Given the significant upregulation of circFIRRE in OS, we assumed that circFIRRE was regulated by upstream transcription factor (TF) in human OS development. We retrieved the promoter sequence of FIRRE from UCUS Genome Browser (http://genome.ucsc.edu/), and then applied three algorithms (UCSC, PROMO, JASPAR) to predict potential TFs that could combine the promoter sequence. It was found that Yin Yang 1 (YY1) was the only TF in the overlap of these algorithms (Fig. 5A). Furthermore, we applied Gene Expression Profiling Interactive Analysis (GEPIA, https://gepia.cancer-pku.cn/) in The Cancer Genome Atlas (TCGA) database and found that the expression level of YY1 and gene FIRRE were elevated in sarcoma (SARC) (Fig. S7A-B). Analysis of our RNA-seq results showed that YY1 and gene FIRRE expressions were elevated in OS samples compared with those in the adjacent no-tumorous samples, which is consistent with the upregulation of circFIRRE (Fig. S7C-D). We then detected expression level of YY1 in 35 paired OS samples and found that circFIRRE was highly expressed in OS samples relative to the adjacent normal controls, and was positively correlated with the expression level of circFIRRE (Fig. 5B-C). At cellular level, YY1 was relatively upregulated in MG63 and U2OS cells compared to hFOB1.19 cells (Fig. 5D).

Subsequently, we compared promoter sequence of circFIRRE with YY1 binding motif in JASPAR and predicted two possible YY1 binding sites in circFIRRE promoter (Fig. 5E), and dual-luciferase reporter assays were applied to verify two possible binding sites. In wild type (WT) group, the luciferase activity of circFIRRE promoter was strengthened under YY1 overexpression, and the mutation of either binding site 1 or 2 partially rescued the strengthened effect, whereas luciferase activity exhibited no significant change when we mutated both binding site 1 and 2 (Fig. 5F).

As the linear transcript of gene FIRRE, linear FIRRE is also a well-known long noncoding RNA (lncRNA) FIRRE. The RNA-seq results showed that the expression level of linear FIRRE in OS samples was more than treble that in adjacent normal samples, but its extent of upregulation was far less than that of circFIRRE (Fig. S7E-F). We next explored the effect of YY1 on the expression change of circFIRRE and linear FIRRE. Three siRNAs of YY1 were designed and si-YY1-1 was selected for following experiments according to the relatively better knockdown effect (Fig. 5G). The expression level of circFIRRE in MG63 and U2OS was decreased significantly after YY1 knockdown, while there was a minimal decreased in linear FIRRE expression, showing no significant difference between the two groups (Fig. 5H-I), YY1 overexpression exhibited the similar results (Fig. 5J-L). These results suggest the probability that the transcriptional regulatory effect of YY1 is mainly on circFIRRE, rather than on linear FIRRE. Altogether, YY1 may be an activator of circFIRRE transcription in OS.

### circFIRRE may sponge miR-486-3p and miR-1225-5p in OS cells

**Fig. 6:**
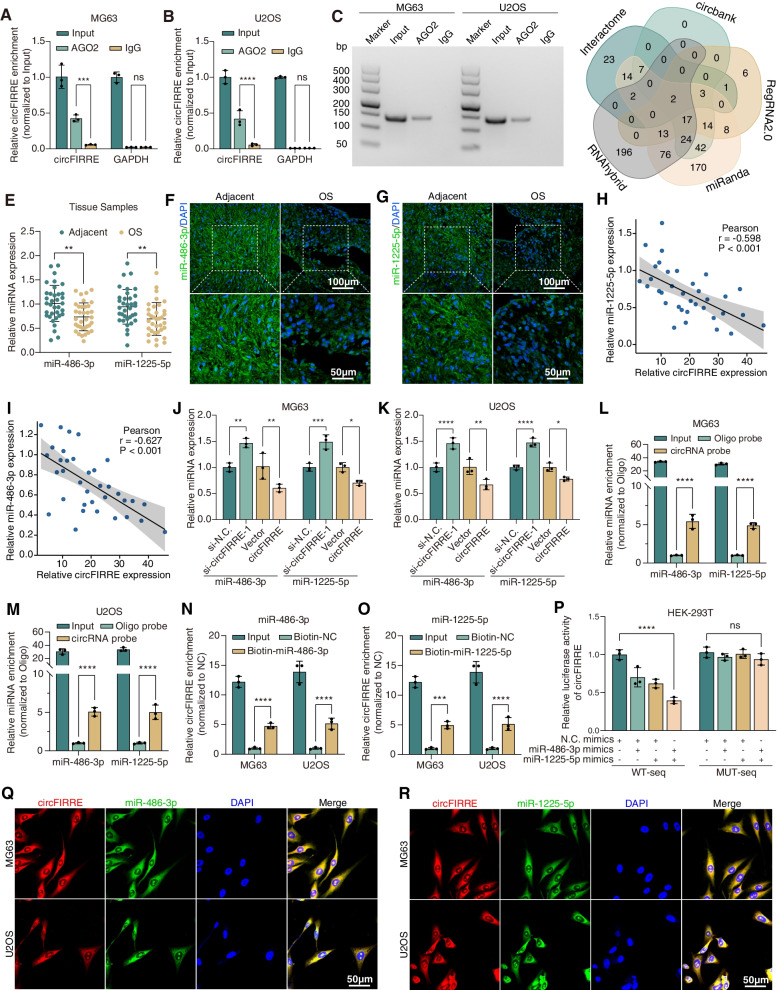
circFIRRE acts as a sponge for miR-486-3p and miR-1225-5p (**A**-**C**) Anti-AGO2 RNA immunoprecipitation (RIP) assay was performed to estimate AGO2 binding to circFIRRE in MG63 and U2OS cells, followed by RT-qPCR and RT-PCR detection, using IgG as the negative control (*n*=3 in each group). **D** Schematic illustration exhibited overlapping of the target miRNAs of circFIRRE predicted by five algorithms. **E** The relative expression of miR-486-3p and miR-1225-5p declined in 35 paired OS samples relative to adjacent normal controls through RT-qPCR detection, respectively. **F**-**G** Representative images of FISH were shown to illustrated the declined miR-486-3p and miR-1225-5p expression in OS samples compare with the adjacent normal samples. Scale bars=100 μm and 50 μm. **H**-**I** Pearson correlation analysis indicated a negative correlation between circFIRRE and two miRNAs in 35 paired samples. **J**-**K** The relative expression of miR-486-3p and miR-1225-5p was detected by RT-qPCR after transfecting circFIRRE siRNA or overexpression vectors in both MG63 and U2OS cells (*n*=3 in each group). **L**-**M** circFIRRE-specific biotin-labelled probe could successfully capture miR-486-3p and miR-1225-5p in MG63 and U2OS, using the oligo probe as the negative control (*n*=3 in each group). **N**-**O** Specific biotin-labelled miR-486-3p and miR-1225-5p probes could successfully capture circFIRRE relative to biotin-NC probe in both MG63 and U2OS (*n*=3 in each group). **P** Dual luciferase assay was performed to estimate the binding between circFIRRE and two miRNAs, after co-transfecting miRNA mimics and luciferase reporter plasmids into HEK-293 T (*n*=3 in each group). **Q**-**R** Typical fields of FISH were showed to illustrate the co-localization of circFIRRE and two miRNAs in both MG63 and U2OS. Scale bar=50 μm. Values are presented as mean ± SD; the bar charts, error bars and dots represent the quantitative analysis of 3 independent experiments; two-way ANOVA were used; *P < 0.05; **P < 0.01; ***P < 0.001; ****P < 0.0001; ns = not significant

Knowing that circFIRRE was predominantly expressed in cytoplasm (Fig. 1F-H), we assumed that circFIRRE might act as miRNA sponge to neutralize miRNA-mediated gene silencing. Initially, RNA immunoprecipitation targeting AGO2 (argonaute RISC catalytic component 2) protein, a core component of RNA-induced silencing complex (RISC) was performed. AGO2 protein acted as an intermediary binding both miRNAs and target mRNAs. The results showed that circFIRRE was enriched specifically by AGO2 pull down instead of immunoglobulin G (IgG) (Fig. 6A-C), suggesting that circFIRRE may bound to RISC and sponge corresponding miRNAs.

Furthermore, we applied five algorithms (miRanda, RNAhybrid, Interactome, circbank and RegRNA2.0) to predict the potential target miRNAs of circFIRRE, and identified miR-486-3p and miR-1225-5p as candidates from the overlap between the databases (Fig. 6D). We sought to verify whether circFIRRE could regulate the expression levels of miR-486-3p and miR-1225-5p by RT-qPCR. The results illustrated that these two miRNAs were significantly declined in 35 paired OS samples and cell lines relative to the adjacent normal controls and osteoblasts (Fig. 6E, Fig. S8A-B), and they were negatively correlated with circFIRRE expression (Fig. 6H-I). FISH assay further confirmed these patterns (Fig. 6F-G). Then, the expression level of miR-486-3p and miR-1225-5p was significantly upregulated by circFIRRE knockdown (si-circFIRRE-1), and down-regulated by circFIRRE overexpression (Fig. 6J-K). These results demonstrated that miR-486-3p and miR-1225-5p were comparatively low-expressed in OS, and negatively regulated by circFIRRE.

Additionally, we investigated whether circFIRRE could directly bind these two miRNAs by pull-down assay by using a specific biotin-labelled circFIRRE probe. It was found that the circFIRRE probe could specifically enrich circFIRRE, miR-486-3p and miR-1225-5p in cell lysate relative to the oligo probe (Fig. S8C-D, Fig. 6L-M). Specific biotin-labelled miR-486-3p and miR-1225-5p probes were applied in pull-down assay for further verification and successfully captured circFIRRE compared with the control probe (Fig. 6N-O). Apart from RNA pull-down, binding sites for miR-486-3p or miR-1225-5p in circFIRRE were forecasted by circinteractome (https://circinteractome.nia.nih.gov/) algorithm (Fig. S8E), and dual-luciferase reporter assay was performed via co-transfecting luciferase reporter plasmids with miR-486-3p or miR-1225-5p mimics into HEK-293 T cells. The results showed that miR-486-3p and miR-1225-5p synergistically diminished the luciferase activity by at least 50% compared with the negative control miRNA. We subsequently mutated the predicted miRNA binding sites from the luciferase reporter plasmid, finding that the mutant luciferase reporter activity remained unchanged after miRNAs transfection (Fig. 6P). These results were supported by the colocalization of circFIRRE and corresponding miRNAs in MG63 and U2OS as sown by double FISH assay (Fig. 6Q-R). All these findings suggest that circFIRRE may work as a sponge for miR-486-3p and miR-1225-5p.

LncRNA FIRRE, as the linear transcript isoform of circFIRRE, can function as a sponge for miRNAs as well [22]. Hence, we needed to ruled out the possibility that FIRRE can sequester miR-486-3p and miR-1225-5p. First, Lncbase, miRcode and miRDB were applied to predict the interactions between FIRRE and two miRNAs (miR-486-3p and miR-1225-5p), and no specific interaction was found between lncFIRRE and miRNAs in three prediction algorithms. Second, we performed FIRRE knockdown and pull-down assays for further verification. In FIRRE knockdown assay, the diminished FIRRE expression level did not affect the expression of the miR-486-3p and miR-1225-5p in both MG63 and U2OS (Fig. S8F-G). In FIRRE pull-down assay, the enrichment of miR-486-3p and miR-1225-5p showed no significant difference between FIRRE probe group and the control (Fig. S8H-J). In all, we could preliminarily rule out the possibility that linear FIRRE can sequester miR-486-3p and miR-1225-5p. Only circFIRRE functioned as a miRNA sponge for miR-486-3p and miR-1225-5p.

### circFIRRE promotes OS tumorigenesis and neovascularization via the miR-486-3p/miR-1225-5p-LUZP1 axis in vitro

**Fig. 7:**
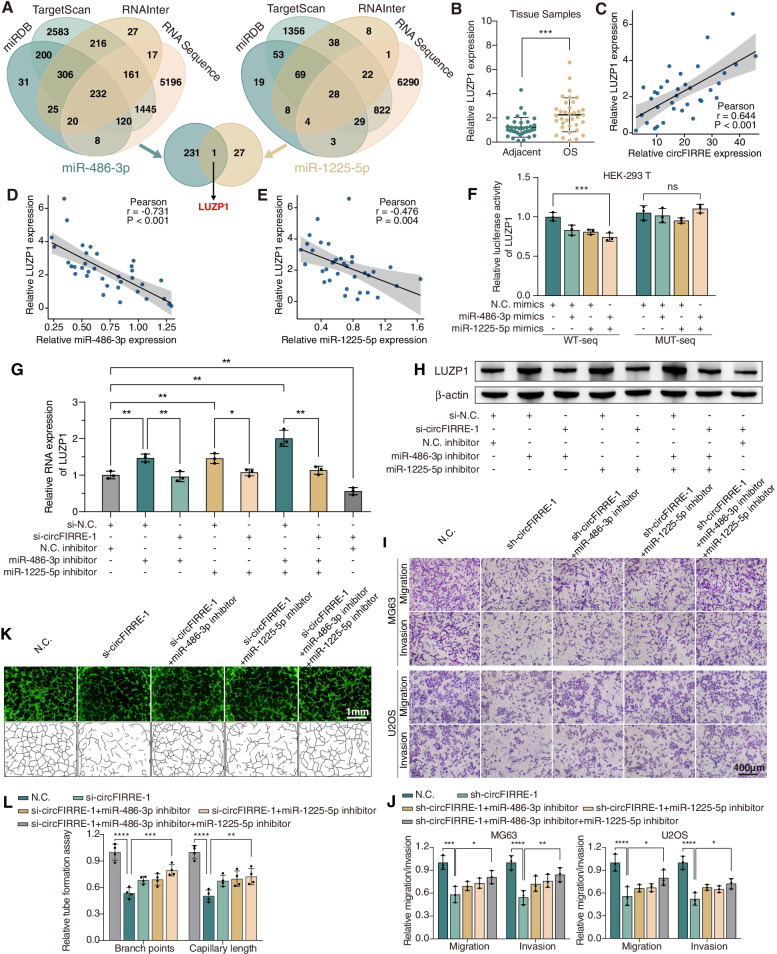
circFIRRE promotes OS tumorigenesis and angiogenesis by miR-486-3p/miR-1225-5p-LUZP1 axis in vitro. **A** Schematic illustration exhibited overlapping of the target genes of miR-486-3p and miR-1225-5p predicted by three algorithms and RNA-sequencing data. **B** The relative expression of LUZP1 declined in 35 paired OS samples relative to adjacent normal controls through RT-qPCR detection, respectively. **C**-**E** Pearson correlation analysis illustrated that LUZP1 was positively correlated with circFIRRE, while negatively correlated with miR-486-3p and miR-1225-5p in 35 paired samples. **F** Dual luciferase assay was performed to verify whether miR-486-3p and miR-1225-5p could bind to predicted binding sites of LUZP1 (*n*=3 in each group). **G**-**H** Relative mRNA and protein expression of LUZP1 were suppressed under circFIRRE silencing, while the downregulation of LUZP1 was retarded by miR-486-3p and (or) miR-1225-5p inhibitors. **I**-**L** Transwell assay and tube formation assay were conducted in MG63, U2OS and HUVEC cells on the third day after co-transfection. Scar bars=400 μm and 1mm. Values are presented as mean ± SD; the bar charts, error bars and dots represent the quantitative analysis of 3 independent experiments; (**B**, **G**, 2-tailed Student t test; **F**, **J**, **L**, two-way ANOVA); *P < 0.05; **P < 0.01; ***P < 0.001; ****P < 0.0001; ns = not significant

It was reported in previous studies that both miR-486-3p and miR-1225-5p were downregulated in OS, and the latter was able to suppress OS progression [23–25]. Thus, we hypothesized that circFIRRE may promote OS progression and neovascularization by restraining the protective effect of two miRNAs and activating downstream gene. In the first place, we aimed to seek the target gene of miR-486-3p and miR-1225-5p in OS. By cross-analyzing three prediction algorithms (miRDB, TargetScan and RNAInter) and our RNA-seq data (fold-change ≥1.5, *p* ≤ 0.05), we found that LUZP1, also known as Leucine Zipper Protein 1, was the sole predicted gene among overlapping gene (Fig. 7A). It was reported that LUZP1 could regulate cancer features by modulating actin cytoskeleton stability and ciliogenesis [26, 27]. Our RNA-seq analysis revealed that LUZP1 was elevated in four paired OS samples compared to normal adjacent samples, data from TCGA database also exhibited that LUZP1 was elevated in sacoma (SARC) compared to the normal control (Fig. S9A-B). The upregulated tendency was then testified in 35 paired clinical samples using RT-qPCR (Fig. 7B), and they were found to be negatively correlated with miR-486-3p and miR-1225-5p expression level, and positively correlated with circFIRRE (Fig. 7C-E).

In the second place, we attempted to verify the role of LUZP1 in tumor progression and angiogenesis. The knockdown effect of LUZP1 specific siRNA was identified in mRNA and protein level (Fig. S9C-D). CCK8 assay, Transwell migration and invasion assays were performed to detect proliferation, migration and invasion abilities of MG63 and U2OS cells transfected with si-LUZP1 or control vector. At the same time, tube formation capabilities of HUVEC cells transfected with si-LUZP1 or control vector were detected using tube formation assay. Results showed that reduction of LUZP1 expression led to weakened proliferation, migration and invasion abilities in OS cells and weakened tube formation capabilities in HUVEC (Fig. S9E-L), indicated that LUZP1 was the target of circFIRRE that affected tumor growth, lung metastasis and angiogenesis.

In the third place, we sought to demonstrate whether circFIRRE regulated LUZP1 via miR-486-3p and miR-1225-5p. Firstly, we co-transfected luciferase reporter plasmids with miR-486-3p and miR-1225-5p mimics in HEK-293 T cells to investigate the interaction between these two miRNAs and LUZP1. The decreased luciferase activity by the LUZP1 3′-UTR was accompanied with miRNAs overexpression. In contrast, luciferase activity remained unchanged compared with the controls after LUZP1 3′-UTR mutation (Fig. 7F, Fig. S10A). Secondly, we detected the regulatory relationship between circFIRRE and LUZP1, and found that the mRNA and protein levels of LUZP1 was strikingly downregulated after circFIRRE knockdown (si-circFIRRE-1 and 2), and significantly upregulated when circFIRRE were overexpressed (Fig. S10B-E). Thirdly, rescue assays were applied using RT-qPCR and Western blot. The results showed that endogenous LUZP1 down-regulation induced by si-circFIRRE-1 was partially rescued by miR-486-3p or miR-1225-5p inhibitors in mRNA and protein level (Fig. 7G-H).

In the last place, we aimed to determine whether circFIRRE promoted tumor progression and angiogenesis in OS via sponging miR-486-3p and miR-1225-5p in vitro. Both circFIRRE stable knockdown OS cells (MG63, U2OS) and untreated HUVEC cells were applied in functional experiments. Functionally, CCK8 assay illustrated that miR-486-3p and miR-1225-5p inhibitors greatly abolished the inhibitory effects on sh-circFIRRE-1 cell proliferation (Fig. S10F). Wound healing assay and Transwell migration and invasion assay illustrated that miR-486-3p and miR-1225-5p inhibitors markedly eliminated the inhibitory effects on migration and invasion in sh-circFIRRE-1 cells (Fig. S10G, Fig. 7I-J). Moreover, tube formation assay revealed that miR-486-3p and miR-1225-5p inhibitors markedly increased the reduced branch points and capillary length in si-circFIRRE-1 cells (Fig. 7K-L). Collectively, these findings demonstrated that circFIRRE promoted OS progression and neovascularization in vitro, at least partially, through miR-486-3p/miR-1225-5p-LUZP1 pathway.

### circFIRRE acts as miRNA sponge to promote both OS tumorigenesis in situ and metastasis in vivo

**Fig. 8:**
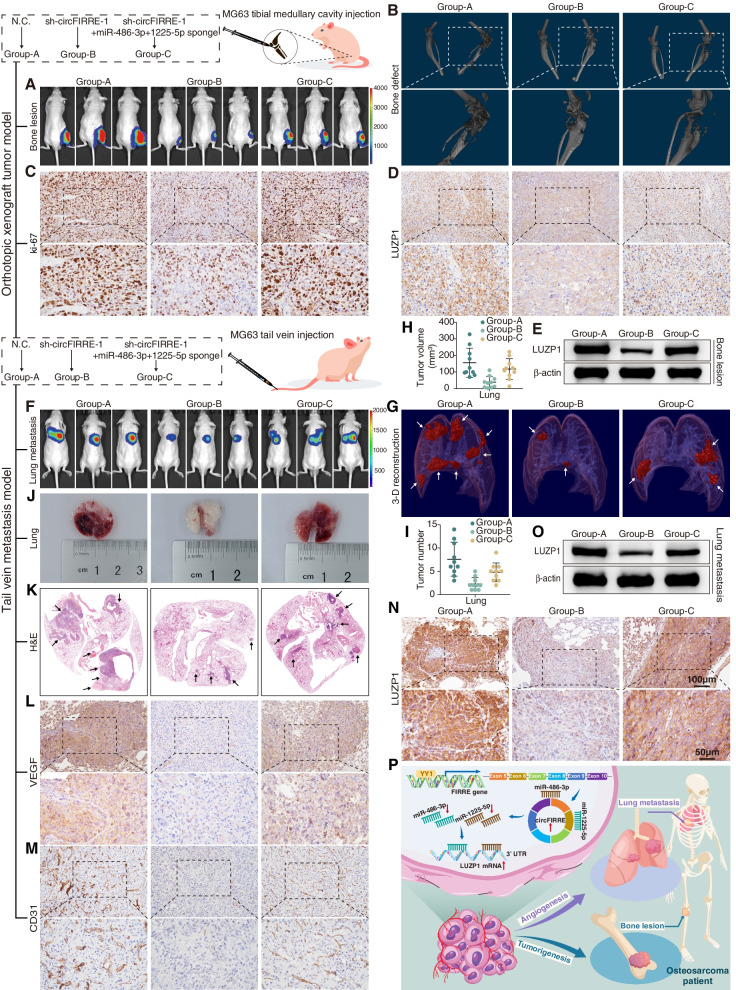
circFIRRE promotes both primary OS progression and metastasis in vivo by sponging miRNAs. MG63 cells infected with empty virus (Group-A), sh-circFIRRE-1 (Group-B) or co-infected with sh-circFIRRE-1 and miR-486-3p+1225-5p sponges (Group-C) for in vivo assays. **A**-**E** Orthotopic xenograft tumor models (*n*=10 in each group). **A** IVIS imaging was applied to determine the OS lesions in situ. The representative images were acquired with the same exposure. **B** Micro-CT scans and 3-D reconstruction were performed to estimate bone destruction induced by tumorigenesis in situ. **C**-**D** Ki-67 and LUZP1 detection in bone lesions by IHC. **E** LUZP1 protein level in bone lesions was examined by western blot. **F**-**N** Tail vein metastasis models (*n*=10 in each group). **F** Representative IVIS imaging of metastatic tumor activity in the lung with the same exposure. **G**-**I** 3-D reconstructions of micro-CT. **G** The white arrowhead indicates the metastatic lesions in the lung. **H**-**I** Quantitation of tumor volumes and numbers. **J**-**K** Collected lungs and Hematoxylin and eosin (H&E) staining. **K** The black arrowhead indicates the metastatic lesions in the lung. **L**-**N** VEGF, CD31 and LUZP1 detection in lung lesions by IHC. **O** LUZP1 protein level in lung lesions was examined by western blot. **P** A proposed model of circFIRRE in modulating OS tumorigenesis, metastasis and angiogenesis via YY1-circFIRRE-miR-486-3p/miR-1225-5p-LUZP1 axis. Scale bars of IHC=100 μm and 50 μm

To verify the functions of circFIRRE-miR-486-3p/miR-1225-5p axis in vivo, both orthotopic xenograft tumor model and tail vein metastasis model were set up. Stable MG63 cells transfected with empty virus were used as negative control (Group-A), stable MG63 cells transfected with sh-circFIRRE-1 (Group-B) or co-transfected with sh-circFIRRE-1 and these two miRNA sponges (Group-C) were established. Considering the tibia is the common primary site of OS, we injected MG63 cells labelled with luminescent dye into the marrow cavity of the right tibia and established an orthotopic xenograft tumor model (ten mice in each group). Four weeks after injection, in vivo bioluminescence imaging (IVIS) assay was applied to monitor the process of tumorigenesis. The luciferase Intensities illustrated that circFIRRE knockdown reduced the tumor size in situ, videlicet, restrained OS cell proliferation, while miR-486-3p and miR-1225-5p suppression alleviated the impairment (Fig. 8A). In addition, micro-CT scans and 3-D reconstruction were performed to evaluate the bone destruction caused by tumorigenesis 5 weeks after injection. The results exhibited grievous tibiofibula and joint destructions in the control group, and the bone destruction was alleviated in sh-circFIRRE group, while the repairment of sh-circFIRRE was rescued by miR-486-3p and miR-1225-5p sponges (Fig. 8B), which is consistent with the consequence of IVIS. Five weeks after injection, mice were sacrificed and OS lesions were obtained for immunohistochemical (IHC) and protein analyses. The IHC results showed that the tumor proliferative activity was diminished as shown by the cell proliferation marker ki-67, and the EMT capacity was inhibited as shown by the mesenchymal markers N-cadherin and Vimentin, and the epithelial marker E-cadherin in sh-circFIRRE-1 group, while these tumor characteristics were rescued by miRNA sponges (Fig. 8C, Fig. S11). The down-regulated protein level of LUZP1 was observed in sh-circFIRRE-1 group relative to the control, while inhibition of miR-486-3p and miR-1225-5p partially abrogated the downregulation in IHC and Western bolt analyses (Fig. 8D-E).

To further investigate how circFIRRE regulated tumor pulmonary metastasis and angiogenesis, we constructed a lung metastasis model by injecting stable MG63 cells labelled with firefly luciferase into the lateral tail vein of nude mice (ten mice in each group). MG63 cells were treated with (Group-B) or without sh-circFIRRE-1 transfection (Group-A) or with both circFIRRE and miR-486-3p & miR-1225-5p silenced (Group-C). According to the luciferase intensity in IVIS assay 4 weeks after injection, we found diffuse metastasis in the lung field of the mice in control group; the lung zone in the sh-circFIRRE group was relatively clear without pervasive metastasis; and silencing miR-486-3p and miR-1225-5p dramatically increased the extent of lung metastasis and the metastatic lesion size compared with sh-circFIRRE group (Fig. 8F). Analogously, micro-CT scans and 3-D reconstruction exhibited a prominent shrinkage in the tumor volume and number in the sh-circFIRRE group relative to the control group, while the volume and number of metastatic lesions was increased in sh-circFIRRE and miRNAs sponge group as compared with sh-circFIRRE group (Fig. 8G-I). The photograph under while light and haematoxylin and eosin (H&E) staining of the excised lungs further confirmed this conclusion (Fig. 8J-K). The expression of circFIRRE and miRNAs was detected by FISH test, and fluorescence in the metastatic lesions displayed the knockdown effect of sh-circFIRRE-1, and the knockdown of circFIRRE was rescued by miR-486-3p and miR-1225-5p sponges (Fig. S12). Vascular endothelial growth factor (VEGF) is an indispensable mediator of angiogenesis, and upregulated expression of VEGF means the course of aberrantly activated angiogenesis [28]. CD31 is the marker of vascular endothelial cells, which represents the formed blood vessel in lesions. VEGF and CD31 staining in metastatic OS showed that angiogenesis was significantly activated in both control and miRNAs sponge groups, and restrained in sh-circFIRRE-1 group (Fig. 8L-M). In addition, IHC demonstrated significant changes in the expression of ki-67, N-cadherin, E-cadherin and Vimentin (Fig. S13). Also, IHC and Western blot validated corresponding changes in LUZP1 expression in the metastatic lesions (Fig. 8N-O). All these findings demonstrated that circFIRRE promoted the in-situ growth, lung metastasis and angiogenesis of OS by sponging miR-486-3p/miR-1225-5p in vivo.

## Discussion

In this study, we screened the anomalously expressed circRNAs between four paired OS and adjacent samples through RNA-seq, and compared their differences in the potential function and latent mechanism of increased circFIRRE expression. The result showed that the transcription factor YY1 at least partially contributed to the upregulation of circFIRRE in human OS.

It is reported [29] that more than 80% high-grade OS patients developed metastasis within one year after amputation treatment, and the lung is the most common site of OS metastasis, comprising more than 85% of distant metastases. Despite the remarkable progress in therapeutic strategies for OS, the curative effect remains unsatisfactory, and the 5-year survival rate remains below 20% in OS patients with lung metastases [2].

Angiogenesis and vascular remodeling promote pulmonary metastasis in that disseminated tumor cells hijack the pre-existent blood vessels of the surrounding lung tissue and tilt the balance toward pro-angiogenesis, and thriving vascular growth further supports secondary lung tumor generation [30–32]. Conspicuous neo-angiogenesis was also reported in OS pulmonary metastasis [33]. In recent years, many anti-angiogenic therapeutic agents have been identified and approved by the US Food and Drug Administration (FDA), including the growth factor inhibitor Bevacizumab (Avastin™), tyrosine kinase inhibitors Apatinib (Aitan®), Sorafenib (Nexavar®), Regorafenib (Stivarga®) and Pazopanib (Armala®), and human recombinant endostatin (Endostar®) [6]. However, researches on the molecular targets proposed for anti-angiogenesis is still in the infantile stage. Clinically, most targeted anti-angiogenic regimens are merely recommended as the second-line agent in OS pulmonary metastasis treatment due to the pessimistic efficacy of long-term administration and potential side effects. The drug resistance to anti-angiogenic reagents severely impairs the long-term therapeutic effect, which is tricky in clinical treatment. Some common side effects have a relative high incidence, including gastrointestinal reaction, delayed wound healing, thrombosis and cardiovascular complications [34].

It was found in our study that elevated expression of circFIRRE promotes the angiogenic capacity of endothelial cells in vitro and pulmonary metastasis in the xenograft tumor model. The expression level of circFIRRE in tumor lesions was positively correlated with the lung metastasis of OS patients. Meanwhile, upregulated circFIRRE facilitated the viability, migration and invasion of OS cells in vitro, and promoted in situ tumorigenesis near the proximal tibia. Analysis of the baseline characteristics of the OS patients revealed that a higher circFIRRE level was correlated with poorer OS and DFS. In conclusion, circFIRRE could be a promising diagnostic or therapeutic target for the following reasons: (i) owing to its covalent cyclic structure, circFIRRE could resist degradation and stably express in OS; (ii) circFIRRE was highly expressed in the OS tissue but not in the adjacent normal tissue, indicating its tissue specificity; (iii) circFIRRE was consistently upregulated in both early stages of primary OS and secondary pulmonary metastatic stage, which may act as an indicator for long-term observation throughout the course of OS development and progression; and(iv) specially, circFIRRE was more highly expressed in the lung metastatic lesions than that in the orthotopic lesions in a mouse xenograft tumor model (Fig. 1C), indicating its temporal specificity.

To date, many strategies have been employed to target circRNAs for therapeutic purposes, including exosome- and nanoparticle- mediated circRNAs delivery therapeutics, cre-lox system-mediated conditional circRNA knockdown and knockout, CRISPR/Cas13-mediated circRNA knockdown, CRISPR/Cas9-mediated circRNA knockout [35, 36]. Many circRNA-targeted therapeutic strategies are currently under preclinical and clinical investigations for efficacy and safety, appearing prospective for clinical application. Deeper investigation on circFIRRE has revealed that it is not only induced by the upstream transcription factor YY1 but regulates the downstream miR-486-3p/miR-1225-5p-LUZP1 pathway, suggesting that it may be possible to use a single-target regimen aiming at circFIRRE for the treatment of pulmonary metastasis in OS. Considering the crosstalk between the upstream and the downstream, a multiple-target regimen by adding YY1 and miRNAs target points on the basis of circFIRRE may be a better therapeutic option in OS patients with advanced lung metastases. In addition, as circFIRRE and relative signaling pathway could promote OS progression and intractable lung metastasis, prophylactic treatment targeting the above target points before symptomatic onset of the disease may have a preventive effect on the occurrence of the disease and can significantly improve the prognosis.

Interestingly, RNA-seq of our study revealed that the level of both circFIRRE and linear FIRRE transcript was elevated in OS samples, while ascensional ranges were highly inconsistent in that circFIRRE was upregulated by 30 times and linear FIRRE was upregulated by 4 times in OS. Physiologically, a previous study [37] reported that dominant isoforms of gene FIRRE transcription product was circular, with merely minor linear FIRRE transcript during human embryonic stem cell differentiation. Pathologically, circFIRRE was prominently upregulated and promoted the progression and multiple organ metastases of multiple carcinomas [38–40], including bladder cancer, breast cancer and non-small cell lung cancer (NSCLC). These results suggest that the increased proportion of circFIRRE in transcriptional outputs may be universal. The circFIRRE-targeted treatment regimen could apply to a wide variety of tumor types, and may ultimately become a broad-spectrum antitumor regimen after further verification. Mechanistically, it was reported that specific repeat sequences in genome, such as Alu elements, could promote circularization in cells [41, 42], so the differences in specific genomic location may partially contribute to the elevated circFIRRE level, but the specific mechanism in transcription imbalance remains to be investigated.

## Conclusions

In conclusion, circFIRRE was upregulated in OS patient samples compared with the adjacent normal samples. Elevated circFIRRE expression was positively corelated with poor clinical characterization. The regulatory effect of the YY1-circFIRRE-miR-486-3p/miR-1225-5p-LUZP1 pathway in OS was preliminarily verified both in vitro and in vivo. Our study is the first to illuminate the role of circFIRRE in tumorigenesis and angiogenesis of OS, which may provide a useful reference for molecular biological, diagnostic and therapeutic research of circRNAs in refractory OS and other neoplasms.

## Supplementary Information

**Additional file 1.** 

**Additional file 2:** **Figure S1.** Validation of differentially expressed circRNAs in RNA-seq. (A) The length distribution of aberrantly expressed circRNAs in RNA-seq. (B) 30 upregulated circRNAs were screened out through filter criteria as described and sorted by name. (C) Quantification of the fluorescence intensity of circFIRRE in FISH assay (*n*=5 in each group). (D) Online prediction algorithms (lncLocator, www.csbio.sjtu.edu.cn/bioinf/lncLocator) was applied to explore the intracellular localization of circFIRRE in OS cells. (E) Quantification of the fluorescence intensity of circFIRRE in both nucleus and cytoplasm in FISH assay (*n*=15 in each group). **Figure S2.** Baseline clinical data and GSEA analysis data. (A) Clinical baseline characteristics of 104 OS patients. (B-F) GSEA (https://www.gsea-msigdb.org/gsea/index.jsp) analysis of hallmark gene sets showed that highly expressed circFIRRE was associated with epithelial mesenchymal transition (EMT), cell cycle (E2F targets, G2M checkpoint and mitotic spindle) and angiogenesis.  **Figure S3.** circFIRRE knockdown can inhibit OS progression in vitro. (A) Three circFIRRE-specific shRNAs were designed and the knockdown efficiency were verified by RT-qPCR in both MG63 and U2OS (*n*=3 in each group). (B) CCK8 assay was applied to estimate cell viability influenced after transient transfection of siRNAs (si-circFIRRE-1 and -2) at different time points in both MG63 and U2OS (*n*=6 at each time point). (C) Wound healing assay exhibited cell migration. Scar bar=200 μm. (D) Schematic of the lentiviral vector GV344 (hU6-MCS-Ubiquitin-firefly_Luciferase-IRES-puromycin). (E) Wound healing assay exhibited cell migration after stable infection of lentivirus in MG63 and U2OS cells. Scar bar=200 μm. (F-H) Flow cytometry analysis of cell cycle distribution. (G-H) Quantification of cell cycle in MG63 and U2OS cells (*n*=3 in each group). Values are presented as mean ± SD; the bar charts, line charts, error bars and dots represent the quantitative analysis of 3 independent experiments; two-way ANOVA were used; *P < 0.05; **P < 0.01; ***P < 0.001; ****P < 0.0001; ns = not significant. **Figure S4.** circFIRRE overexpression can promote OS progression in vitro. (A) Schematic of the circFIRRE overexpression vector GM-7183. (B) Sanger sequencing was applied to verify back-spliced junction of circFIRRE (GGAG) after vector construction. (C) Wound healing assay exhibiting cell migration. Scar bar=200 μm. (D-F) Flow cytometry assays presenting cell cycle distribution. Values are presented as mean ± SD; the bar charts, error bars and dots represent the quantitative analysis of 3 independent experiments; two-way ANOVA were used; *P < 0.05; **P < 0.01. **Figure S5.** circFIRRE knockdown can inhibit angiogenesis. (A) CCK8 assay was applied to estimate cell viability influenced after transient transfection of siRNAs (si-circFIRRE-1 and -2) at different time points in HUVEC cells (*n*=6 at each time point). (B-C) EdU assay was performed to estimate cell proliferation after circFIRRE silencing in HUVEC cells. S-phase entry is visualized by EdU incorporation (green); DAPI-stained nuclei (blue). Scar bar=200 μm. (C) Quantification was conducted as described (*n*=5 in each group). (D-F) Wound healing and Tranwell migration assays exhibited cell migration after circFIRRE silencing in HUVEC cells. Scar bars=200 μm and 400 μm. (F) Quantification of Transwell assay was conducted as described (*n*=5 in each group). (G) White light micrographs (top) and highlighted microvessel areas (red) in fluorescence micrographs (bottom) at the same fields of Figure 4G. Scar bars=1 mm and 400 μm. (H) Quantification of aortic ring microvessel area compared to negative control aorta (*n*=5 in each group). (I) White light images of CAM photographed in fertilized eggs at the same fields of Figure 4H. (J) The statistical results of the CAM assay (*n*=5 in each group). Values are presented as mean ± SD; the bar charts, line charts, error bars and dots represent the quantitative analysis of 3 independent experiments; (C, F, H, one-way ANOVA; J, two-way ANOVA); *P < 0.05; **P < 0.01; ***P < 0.001; ****P < 0.0001. **Figure S6.** circFIRRE overexpression can promote angiogenesis. (A) Relative circFIRRE expression was detected by RT-qPCR after overexpression vectors or scramble vectors transfection in HUVEC cells (*n*=3 in each group). (B-D) CCK8 and EdU assays exhibited cell proliferation in HUVEC under circFIRRE overexpression. Scar bar=200 μm. (E-G) Wound healing and Transwell migration assays exhibited cell migration in HUVEC under circFIRRE overexpression. Scar bars=200 μm and 400 μm. (H) White light micrographs (top) and highlighted microvessel areas (red) in fluorescence micrographs (bottom) at the same fields of Figure 4K. Scar bars=1mm and 400 μm. (I) Quantification of aortic ring microvessel area compared to negative control aorta (*n*=5 in each group). (J) White light images of CAM photographed in fertilized eggs at the same fields of Figure 4L. (K) The statistical results of the CAM assay (*n*=5 in each group). Values are presented as mean ± SD; the bar charts, line charts, error bars and dots represent the quantitative analysis of 3 independent experiments; (A, D, G, I, 2-tailed Student t test; B, K, two-way ANOVA); *P < 0.05; **P < 0.01; ****P < 0.0001. **Figure S7.** circFIRRE is induced by YY1. (A-B) Relative expression of YY1 and gene FIRRE was upregulated in sarcoma (SARC) in TCGA database. (C-E) The FPKM of YY1, gene FIRRE and linear FIRRE in RNA-seq. (F) Fold change of linear FIRRE and circFIRRE in RNA-seq.  **Figure S8.** circFIRRE can sponge miR-486-3p and miR-1225-5p. (A-B) Relative expression of miR-486-3p and miR-1225-5p was examined by RT-qPCR in 5 OS cell lines and normal osteoblasts (*n*=3 in each group). (C-D) Preliminary experiments to test circFIRRE probe by RT-qPCR and RT-PCR in both MG63 and U2OS cells (*n*=3 in each group). (E) The predicted binding sites of miR-486-3p and miR-1225-5p in circFIRRE. (F) Validation of FIRRE siRNAs knockdown in MG63 and U2OS (*n*=3 in each group). (G) Relative expression of miR-486-3p and miR-1225-5p was examined by RT-qPCR under FIRRE knockdown (*n*=3 in each group). (H-I) Preliminary experiments to test FIRRE probe by RT-qPCR and RT-PCR in both MG63 and U2OS cells (*n*=3 in each group). (J) Relative expression of miR-486-3p and miR-1225-5p was examined by RT-qPCR under FIRRE pull-down (*n*=3 in each group). Values are presented as mean ± SD; the bar charts, error bars and dots represent the quantitative analysis of 3 independent experiments in A, B; (A, B, one-way ANOVA; C, F, G, H, J, two-way ANOVA); **P < 0.01; ***P < 0.001; ****P < 0.0001. **Figure S9.** LUZP1 knockdown can inhibit tumor proliferation, progression and angiogenesis. (A) The FPKM of LUZP1 in RNA-seq. (B) Relative expression of LUZP1 in sarcoma (SARC) in TCGA database. (C-D) Validation of LUZP1 siRNA knockdown in MG63 and U2OS. (E-F) CCK8 assay was applied to estimate cell viability influenced by LUZP1 knockdown at different time points in both MG63 and U2OS (*n*=6 at each time point). (G-J) Transwell migration and invasion assays were employed to detect cell migration and invasion abilities influenced by LUZP1 knockdown (*n*=3 in each group). Scar bar=400 μm. (K-L) Tube formation assay was applied to determine cell tube formation ability influenced by LUZP1 knockdown in HUVEC cells. Scar bar=1mm. Values are presented as mean ± SD; the bar charts, line charts, error bars and dots represent the quantitative analysis of 3 independent experiments; two-way ANOVA were used; *P < 0.05; **P < 0.01; ***P < 0.001; ****P < 0.0001.** Figure S10.** circFIRRE promotes OS tumorigenesis by miR-486-3p/miR-1225-5p-LUZP1 axis in vitro. (A) The predicted binding sites of miR-486-3p and miR-1225-5p in LUZP1. (B-E) The mRNA and protein level of LUZP1 after circFIRRE silencing or overexpression. (F) CCK8 assay exhibited cell proliferation in MG63 and U2OS cells (*n*=3 in each group). (G) Wound healing assay exhibited cell migration in MG63 and U2OS cells. Scar bar=200 μm. Values are presented as mean ± SD; the bar charts, line charts, error bars and dots represent the quantitative analysis of 3 independent experiments; two-way ANOVA were used; *P < 0.05; **P < 0.01; ***P < 0.001; ****P < 0.0001. **Figure S11.** Representative IHC staining of primary OS lesions in xenograft models (E-cadherin, N-cadherin and Vimentin). Scar bars=100 μm and 50 μm. **Figure S12.** Representative FISH images of lung metastatic lesions exhibited circFIRRE, miR-486-3p and miR-1225-5p expression in xenograft models. Scar bar=100 μm. **Figure S13.** Representative IHC staining of lung metastatic lesions in xenograft models (ki-67, E-cadherin, N-cadherin and Vimentin). Scar bars=100 μm and 50 μm. 

**Additional file 3:** **Table S1.** 163 differentially expressed circRNAs met the screening criteria in RNA sequencing. 

## Data Availability

The datasets used and/or analysed during the current study are available from the corresponding author on reasonable request.

## Abbreviations

OS: osteosarcoma
circRNA: circular RNA
Yin Yang 1: YY1
LUZP1: Leucine Zipper Protein 1
RNA-seq: RNA-sequencing
RT-qPCR: quantitative real-time polymerase chain reaction
FIRRE: Firre Intergenic Repeating RNA Element
RNase R: ribonuclease R
AGE: agarose gel electrophoresis
OS: overall survival
DFS: disease-free survival
GSEA: gene-set enrichment analysis
EMT: epithelial mesenchymal transition
CAM: chick chorioallantoic membrane
TF: transcription factor
GEPIA: Gene Expression Profiling Interactive Analysis
TCGA: The Cancer Genome Atlas
WT: wild type
Ago2: argonaute RISC catalytic component 2
RISC: RNA-induced silencing complex
IgG: immunoglobulin G
IVIS: in vivo bioluminescence imaging
IHC: immunohistochemical
H&E: haematoxylin and eosin
VEGF: Vascular endothelial growth factor
FISH: Fluorescent In Situ Hybridization

## References

[1] Gill J, Gorlick R (2021). Advancing therapy for osteosarcoma. Nature reviews Clinical oncology.

[2] Meltzer P, Helman L (2021). New Horizons in the Treatment of Osteosarcoma. The New England journal of medicine.

[3] Bielack S, Kempf-Bielack B, Delling G, Exner G, Flege S, Helmke K, Kotz R, Salzer-Kuntschik M, Werner M, Winkelmann W, et al. Prognostic factors in high-grade osteosarcoma of the extremities or trunk: an analysis of 1,702 patients treated on neoadjuvant cooperative osteosarcoma study group protocols. Journal of clinical oncology : official journal of the American Society of Clinical Oncology. 2002;20:776–90. 10.1200/JCO.2002.20.3.776 11821461

[4] Zambo I, Veselý K (2014). WHO classification of tumours of soft tissue and bone 2013: the main changes compared to the 3rd edition. Cesk Patol.

[5] Kreuter M, Bieker R, Bielack S, Auras T, Buerger H, Gosheger G, Jurgens H, Berdel W, Mesters R. Prognostic relevance of increased angiogenesis in osteosarcoma. Clinical cancer research : an official journal of the American Association for Cancer Research. 2004;10:8531–7. 10.1158/1078-0432.CCR-04-0969 15623635

[6] Liu Y, Huang N, Liao S, Rothzerg E, Yao F, Li Y, Wood D, Xu J: Current research progress in targeted anti-angiogenesis therapy for osteosarcoma. In Cell proliferation, vol. 54. pp. e131022021:e13102. 10.1111/cpr.13102 PMC8450128 34309110

[7] Hanahan D, Weinberg R (2011). Hallmarks of cancer: the next generation. Cell.

[8] Assi T, Watson S, Samra B, Rassy E, Le Cesne A, Italiano A, Mir O: Targeting the VEGF Pathway in Osteosarcoma. Cells 2021, 10. 10.3390/cells10051240 PMC8157846 34069999

[9] Xie L, Ji T, Guo W (2017). Anti-angiogenesis target therapy for advanced osteosarcoma (Review). Oncol Rep.

[10] Kristensen LS, Andersen MS, Stagsted LVW, Ebbesen KK, Hansen TB, Kjems J (2019). The biogenesis, biology and characterization of circular RNAs. Nat Rev Genet.

[11] S M, M J, A E, F T, J K, A R, L M, SD M, LH G, M M, et al: Circular RNAs are a large class of animal RNAs with regulatory potency. Nature 2013, 495:333-338. 10.1038/nature11928 23446348

[12] Gomes CPC, Schroen B, Kuster GM, Robinson EL, Ford K, Squire IB, Heymans S, Martelli F, Emanueli C, Devaux Y (2020). Regulatory RNAs in Heart Failure. Circulation.

[13] Lei M, Zheng G, Ning Q, Zheng J, Dong D (2020). Translation and functional roles of circular RNAs in human cancer. Mol Cancer.

[14] Smid M, Wilting SM, Uhr K, Rodríguez-González FG, de Weerd V, Prager-Van der Smissen WJC, van der Vlugt-Daane M, van Galen A, Nik-Zainal S, Butler A, et al: The circular RNome of primary breast cancer. Genome Res 2019, 29:356-366. 10.1101/gr.238121.118 PMC6396421 30692147

[15] Chen J, Liu G, Wu Y, Ma J, Wu H, Xie Z, Chen S, Yang Y, Wang S, Shen P (2019). CircMYO10 promotes osteosarcoma progression by regulating miR-370-3p/RUVBL1 axis to enhance the transcriptional activity of β-catenin/LEF1 complex via effects on chromatin remodeling. Mol Cancer.

[16] Liu G, Huang K, Jie Z, Wu Y, Chen J, Chen Z, Fang X, Shen S (2018). CircFAT1 sponges miR-375 to promote the expression of Yes-associated protein 1 in osteosarcoma cells. Mol Cancer.

[17] Shen S, Yao T, Xu Y, Zhang D, Fan S, Ma J (2020). CircECE1 activates energy metabolism in osteosarcoma by stabilizing c-Myc. Molecular cancer.

[18] Wu Y, Xie Z, Chen J, Chen J, Ni W, Ma Y, Huang K, Wang G, Wang J, Ma J (2019). Circular RNA circTADA2A promotes osteosarcoma progression and metastasis by sponging miR-203a-3p and regulating CREB3 expression IF; 10. Mol Cancer.

[19] Yu G, Wang LG, Han Y, He QY (2012). clusterProfiler: an R package for comparing biological themes among gene clusters. Omics.

[20] Glažar P, Papavasileiou P, Rajewsky N (2014). circBase: a database for circular RNAs. Rna.

[21] Lin Y, Pan X, Shen HB: lncLocator 2.0: a cell-line-specific subcellular localization predictor for long non-coding RNAs with interpretable deep learning. Bioinformatics 2021. 10.1093/bioinformatics/btab127 33630066

[22] Wang S, Wang Y, Wang S, Tong H, Tang Z, Wang J, Zhang Y, Ou J, Quan Z (2021). Long Non-coding RNA FIRRE Acts as a miR-520a-3p Sponge to Promote Gallbladder Cancer Progression via Mediating YOD1 Expression. Frontiers in genetics.

[23] Zhang W, Wei L, Sheng W, Kang B, Wang D, Zeng H (2020). miR-1225-5p Functions as a Tumor Suppressor in Osteosarcoma by Targeting Sox9. DNA and cell biology.

[24] Gong Y, Wei Z, Liu J (2021). MiRNA-1225 Inhibits Osteosarcoma Tumor Growth and Progression by Targeting YWHAZ. OncoTargets and therapy.

[25] Monterde-Cruz L, Ramírez-Salazar E, Rico-Martínez G, Linares-González L, Guzmán-González R, Delgado-Cedillo E, Estrada-Villaseñor E, Valdés-Flores M, Velázquez-Cruz R, Hidalgo-Bravo A (2020). MicroRNA expression in relation with clinical evolution of osteosarcoma. Pathology, research and practice.

[26] Bozal-Basterra L, Gonzalez-Santamarta M, Muratore V, Bermejo-Arteagabeitia A, Da Fonseca C, Barroso-Gomila O, Azkargorta M, Iloro I, Pampliega O, Andrade R, et al: LUZP1, a novel regulator of primary cilia and the actin cytoskeleton, is a contributing factor in Townes-Brocks Syndrome. eLife 2020, 9. 10.7554/eLife.55957 PMC7363444 32553112

[27] Gonçalves J, Sharma A, Coyaud É, Laurent E, Raught B, Pelletier L: LUZP1 and the tumor suppressor EPLIN modulate actin stability to restrict primary cilia formation. The Journal of cell biology 2020, 219. 10.1083/jcb.201908132 PMC7337498 32496561

[28] Sullivan LA, Brekken RA (2010). The VEGF family in cancer and antibody-based strategies for their inhibition. MAbs.

[29] Bielack SS, Kempf-Bielack B, Delling G, Exner GU, Flege S, Helmke K, Kotz R, Salzer-Kuntschik M, Werner M, Winkelmann W (2002). Prognostic factors in high-grade osteosarcoma of the extremities or trunk: an analysis of 1,702 patients treated on neoadjuvant cooperative osteosarcoma study group protocols. J Clin Oncol.

[30] Weis S, Cheresh D (2011). Tumor angiogenesis: molecular pathways and therapeutic targets. Nature medicine.

[31] Kuczynski E, Vermeulen P, Pezzella F, Kerbel R, Reynolds A (2019). Vessel co-option in cancer. Nature reviews Clinical oncology.

[32] Altorki N, Markowitz G, Gao D, Port J, Saxena A, Stiles B, McGraw T, Mittal V (2019). The lung microenvironment: an important regulator of tumour growth and metastasis. Nature reviews Cancer.

[33] Ségaliny A, Mohamadi A, Dizier B, Lokajczyk A, Brion R, Lanel R, Amiaud J, Charrier C, Boisson-Vidal C, Heymann D (2015). Interleukin-34 promotes tumor progression and metastatic process in osteosarcoma through induction of angiogenesis and macrophage recruitment. International journal of cancer.

[34] Cook K, Figg W: Angiogenesis inhibitors: current strategies and future prospects. CA: a cancer journal for clinicians 2010, 60:222-243. 10.3322/caac.20075 PMC2919227 20554717

[35] He A, Liu J, Li F, Yang B (2021). Targeting circular RNAs as a therapeutic approach: current strategies and challenges. Signal transduction and targeted therapy.

[36] Winkle M, El-Daly S, Fabbri M, Calin G (2021). Noncoding RNA therapeutics - challenges and potential solutions. Nature reviews Drug discovery.

[37] Izuogu O, Alhasan A, Mellough C, Collin J, Gallon R, Hyslop J, Mastrorosa F, Ehrmann I, Lako M, Elliott D (2018). Analysis of human ES cell differentiation establishes that the dominant isoforms of the lncRNAs RMST and FIRRE are circular. BMC genomics.

[38] Jin M, Lu S, Wu Y, Yang C, Shi C, Wang Y, Huang G (2020). Hsa_circ_0001944 promotes the growth and metastasis in bladder cancer cells by acting as a competitive endogenous RNA for miR-548. J Exp Clin Cancer Res.

[39] Fu B, Liu W, Zhu C, Li P, Wang L, Pan L, Li K, Cai P, Meng M, Wang Y (2021). Circular RNA circBCBM1 promotes breast cancer brain metastasis by modulating miR-125a/BRD4 axis. International journal of biological sciences.

[40] Dou Y, Tian W, Wang H, Lv S (2021). Circ_0001944 Contributes to Glycolysis and Tumor Growth by Upregulating NFAT5 Through Acting as a Decoy for miR-142-5p in Non-Small Cell Lung Cancer. Cancer management and research.

[41] Zhang X, Wang H, Zhang Y, Lu X, Chen L, Yang L (2014). Complementary sequence-mediated exon circularization. Cell.

[42] Liang D, Wilusz J (2014). Short intronic repeat sequences facilitate circular RNA production. Genes & development.

