# Supplementary material for: Circular RNA circFIRRE drives osteosarcoma progression and metastasis through tumorigenic-angiogenic coupling
Source: Mol Cancer. 2022 Aug 19;21:167. doi: 10.1186/s12943-022-01624-7 (PMC9389772; doi:10.1186/s12943-022-01624-7)
Supplement: Supplementary file 1 — Additional file 1. [file 12943_2022_1624_MOESM1_ESM.docx]

**Circular RNA circFIRRE drives** **osteosarcoma progression and metastasis through** **tumorigenic-angiogenic coupling**

***Additional file 1***

***Supplementary*** ***materials and methods***

Lingfeng Yu, Hao Zhu, Zhen Wang, Jianhao Huang, Yan Zhu, Gentao Fan, Yicun Wang, Xi Chen*, Guangxin Zhou*

Table of contents

Supplementary materials and method----------------------------------------------------------2

Primers, probes, siRNAs and shRNAs -------------------------------------------------------17

**Supplementary materials and methods**

**RNA-sequencing**

Total RNA was isolated and purified using TRIzol reagent (Invitrogen, Carlsbad, CA, USA) following the manufacturer's procedure. The RNA amount and purity of each sample was quantified using NanoDrop ND-1000 (NanoDrop, Wilmington, DE, USA). The RNA integrity was assessed by Bioanalyzer 2100 (Agilent, CA, USA) with RIN number >7.0, and confirmed by electrophoresis with denaturing agarose gel. Approximately 2ug of total RNA was used to remove ribosomal RNA according to the manuscript of the Epicentre Ribo-Zero Gold Kit (Illumina, San Diego, USA). Following purification, the ribo-minus RNA was fragmented into small pieces using Magnesium RNA Fragmentation Module (NEB, cat.e6150, USA) under 94℃ 5-7min. Then the cleaved RNA fragments were reverse-transcribed to create the cDNA by SuperScript™ II Reverse Transcriptase (Invitrogen, cat. 1896649, USA), which were next used to synthesise U-labeled second-stranded DNAs with E. coli DNA polymerase I (NEB, cat.m0209, USA), RNase H (NEB, cat.m0297, USA) and dUTP Solution (Thermo Fisher, cat.R0133, USA) . An A-base is then added to the blunt ends of each strand, preparing them for ligation to the indexed adapters. Each adapter contains a T-base overhang for ligating the adapter to the A-tailed fragmented DNA. Single- or dual-index adapters are ligated to the fragments, and size selection was performed with AMPureXP beads. After the heat-labile UDG enzyme (NEB, cat.m0280, USA) treatment of the U-labeled second-stranded DNAs, the ligated products are amplified with PCR by the following conditions: initial denaturation at 95℃ for 3 min; 8 cycles of denaturation at 98℃ for 15 sec, annealing at 60℃ for 15 sec, and extension at 72℃ for 30 sec; and then final extension at 72℃ for 5 min. The average insert size for the final cDNA library was 300±50 bp. At last, we performed the 2×150bp paired-end sequencing (PE150) on an Illumina HiSeq 4000 sequencing system (Aksomics Inc, Shanghai, China) following the vendor's recommended protocol.

**Gene-set enrichment analysis (GSEA)**

GSEA analysis strategies has been described in detail in this article [1]. Enrichment analysis was conducted using our RNA-sequencing data of OS tissues and adjacent normal tissues. Gene sets were set to hallmark gene sets from MSigDB (<https://www.gsea-msigdb.org/gsea/msigdb/index.jsp>), and gene sets with adjusted p < 0.05 and FDR < 0.25 were considered significantly enriched. Enrichment analysis was performed using clusterProfiler package [2].

**Cell lines and cultures**

HEK-293 T cells (ATCC: CRL-1573), human umbilical vein endothelial cell line HUVEC (ATCC: CRL-1730), HUVEC-derived cell line EA.hy926 (ATCC: CRL-2922), human dermal microvascular endothelia cell line HMEC-1 (ATCC: CRL-3243), and human OS cell lines, including MG63 (ATCC: CRL-1427TM), U2OS (ATCC: HTB-96TM), HOS (ATCC: CRL-1543), SJSA-1 (ATCC: CRL-2098), 143B (ATCC: CRL-8303) and human osteoblast hFOB1.19 cells (ATCC: CRL-11372) were purchased from the ATCC (Manassas, VA, USA). Mycoplasma infection was ruled out in all cell lines using the Venor GeM Mycoplasma Detection Kit (Minerva Biolabs, Berlin, Germany). HUVEC cells were maintained in Endothelial cell medium (ECM, ScienCell, USA) supplemented with 5% fetal bovine serum (ScienCell, USA) and endothelial cell growth supplement (ECGS, ScienCell, USA). Ea.hy926, HEK-293 T cells were maintained in Dulbecco's Modified Eagle's Medium (DMEM) supplemented with 10% fetal bovine serum (Gibco, Grand Island, NY, USA). HMEC-1 cells were maintained in MCDB131 medium supplemented with 10 ng/ml epidermal growth factor, 1 µg/ml hydrocortisone, 10 mM glutamine, and 10% fetal bovine serum. HOS, 143B, MG63 cells were maintained in Eagle's Minimum Essential Medium (EMEM) supplemented with 10% fetal bovine serum. U2OS cells were maintained in McCoy's 5a Modified Medium supplemented with 10% fetal bovine serum. SJSA-1 cells were maintained in RPMI-1640 Medium supplemented with 10% fetal bovine serum. hFOB1.19 cells were maintained in Dulbecco’s modified Eagle’s medium/Nutrient Mixture F-12 (DMEM/F-12) supplemented with 0.3 mg/ml G418 (Invitrogen, Carlsbad, CA, USA) and 10% fetal bovine serum. hFOB1.19 cells were incubated at 5% CO2 at 34 °C, remaining cells were cultured at 5% CO2 at 37 °C.

**Nucleocytoplasmic separation**

The PARIS™ kit (Invitrogen, AM1921) was applied for the nuclear and cytoplasmic RNA separation experiment. Briefly, 5 × 10^6^ cells (MG63 or U2OS) were collected and resuspended in 500 μl Cell Fractionation Buffer and incubated on ice for 10 min followed by homogenization. After centrifugation at 400×g for 5 min, the cytoplasmic fraction was obtained in the supernatant. The pellet was then resuspended in 500 μl Cell Disruption Buffer to obtain the nuclear fraction. The RNA from nuclear and cytoplasmic fractions were obtained through Filter Cartridge and resuspended in the same volume of Elution Solution.

For circFIRRE and linear FIRRE expression determination, the same volume of RNA (the same volume of Elution Solution) from each fraction were subject to RT-qPCR. To verify the nuclear and cytoplasmic fractionation of the mRNA, U6 and GAPDH were used as controls, respectively. For the relative quantification of each fraction the following formula was used:

(i) Nuclear %=2^ –nuclear Ct value / (2^- cytoplasmic Ct value + 2^- nuclear Ct value)

(ii) Cytoplasmic %=1- Nuclear %

**Plasmids, siRNAs, transient transfection and stable infection**

circFIRRE sequence was amplified via specific primers and reassembles into a GM-7183 (PGMLV-circRNA_Mini-GFP-Puro) overexpressed vector via restriction enzyme sites Bsu15I and HpaI. For circFIRRE knockdown, three pairs of siRNAs targeting the back-spliced junction site were designed, the sequence were listed in **Additional file 1 Page 17**. Both overexpression vector and siRNAs were purchased from Genomeditech (Shanghai, China). The transient transfection of plasmids and siRNAs was conducted using Lipofectamine 3000 (Invitrogen, Carlsbad, CA, USA) in Opti-MEM.

For stable infection, lentivirus-sh-circFIRRE and lentivirus-miRNA sponges were synthesized by GeneChem (Shanghai, China) using the GV344 vector (hU6‐MCS‐Ubiquitin‐firelfy_Luciferase-IRES‐puromycin). The scrambled GV344 vector (TTCTCCGAACGTGTCACGT) was applied for negative control. Lentiviruses were employed to infect cells in appropriate virus titer, complying with the manufacturer’s instructions. 36 hours after lentiviruses infection, the cells were screened in puromycin (Gibco, Grand Island, NY, USA) concentration gradient from 2μg/ml to 10μg/ml for 2 weeks, and viable cells were elected for stable cell strains.

**Sanger sequencing**

Sanger sequencing (Genewiz Inc., Suzhou, China) was used as described [3] to verify the presence of the circFIRRE-specific backsplice junction (GGAG).

For circFIRRE identification, the PCR products amplified using circFIRRE specific primers were detected by agarose gel electrophoresis, the qualified PCR products with correct length were purified using FastPure Gel DNA Extraction Mini Kit (Vazyme, China). Then the purified products were used for Sanger sequencing.

For circFIRRE overexpression plasmid identification, plasmid DNA was extracted using E.Z.N.A endo-free plasmid DNA mini kit (Omega Bio-Tek, Norcross, USA) and verified using Sanger sequencing.

**Quantitative reverse-transcription polymerase chain reaction (RT-qPCR) and Western blot**

Total RNA was isolated from various tissues and cell lines using TRIzol (Invitrogen, Carlsbad, CA, USA). For circRNA and mRNA analyses, HiScript II Q Select RT SuperMix and ChamQ Universal SYBR qPCR Master Mix were purchased from Vazyme (Nanjing, China), GAPDH was served as the internal reference. For miRNA detections, TaqMan miRNA probes (Applied Biosystems, CA, USA) for miR-486-3p, miR-1225-5p and U6 were applied according to the manufacturer’s instructions, in which U6 was chosen as the internal reference. Subsequently reaction and quantitative analysis was performed using a LightCycler96 System (Roche, IN, USA). The relative fold-change was analyzed by the 2^-△△CT^ method. Primers used for RT-qPCR are listed below.

For western blot analysis, protein was extracted from ultracentrifugation following cell lysis using RIPA Lysis Buffer (Beyotime, Shanghai, China) on ice. After quantified by a Pierce BCA Protein Assay kit (Thermo Scientific, CA, USA), protein was electrophoretically separated and transferred onto polyvinylidene fluoride (PVDF) membranes (Millipore, Billerica, USA). The membranes were blocked for 15 min with Blocking Buffer for Western Blot (Beyotime, Shanghai, China) and incubated overnight at 4 ℃ with anti-LUZP1 antibody (1:1000, proteintech, USA), anti-β-actin antibody (1:1000, Cell Signaling Technology, USA). After washings, a secondary antibody (1:2000, Cell Signaling Technology, USA) was incubated for 1 hour followed by another washes. Membranes were incubated in Dura ECL kit (Fudebio, Hangzhou, China) and visualized by a chemiluminescence system (Tanon, Shanghai, China).

**Ribonuclease R (RNase R) assay and Nucleic acid** **electrophoresis**

RNase R assay was conducted as previously described [4]. 2 μg of total RNA was incubated for 15 min at 37 ℃ with or without 3 U/μg RNase R (Geneseed, Guangzhou, China). Then the specific divergent primers for circFIRRE and linear FIRRE were applied for amplification after RNase R treatment. GAPDH was employed as a control for linear mRNA digestion in RNase R treatment. The Ct value of GAPDH for mock treatment was used for both RNase R-treated and mock-treated samples due to degradation of linear RNAs in the RNase R-treated samples.

For nucleic acid electrophoresis, amplified products from either cDNA or gDNA were separated in 2% agarose gel at 120 V for 30 min, and examined in a gel imaging system (Tanon, Shanghai, China).

**Actinomycin D assay**

OS cells were equally seeded in 5 wells in 6-well cell culture plates (5 × 10^4^ cells per well). The second day, actinomycin D (5 μg/ml, Leagene, Beijing, China) was spectively added for different time gradients (0, 4, 8, 12, 24 h). The total RNA was extracted from cells in different point in times, and the expression level of circFIRRE and linear FIRRE were analyzed by RT-qPCR and normalized to the control group (0 h group).

**Cell Counting Kit (CCK)-8 assay**

For CCK8 assay, cells were equally seeded in 96-well cell culture plates (3 × 10^3^ cells per well). At different point of time (0, 24, 48, 72 h and 96 h), 100 μL complete medium containing 10 μL CCK8 (Yeasen, Shanghai, China) was added to culture plates and incubated for 1 h. The absorbance at 450 nm was detected in a microplate reader (Thermo Scientific, CA, USA).

**5-Ethynyl-2′-deoxyuridine (EdU) assay**

EdU assay kit was purchased from RibiBio (Guangzhou, China) for EdU assays. Cells were seeded into 96-well cell culture plates overnight. In the second day, we added EdU solution (25 μM) to complete medium and incubated for recommended time. Then cells in culture plates were fixed by paraformaldehyde for 2 h and permeabilized by 0.5% TritonX-100 for 10 min. Apollo reaction solution (200 μL) and DAPI (200 μL) were added to stain EdU and nuclei respectively for 30 min. Luminescence was photographed using an Olympus IX 71 inverted fluorescence microscope (Olympus, Tokyo, Japan) to analyze cell proliferation and DNA synthesis. For image quantification, at least 5 randomly selected images were chosen from each independent experiment, and the mean level of 3 independent experiment were quantified at last.

**Wound healing assay**

We cultured cells in 6-well cell culture plates until 100% confluence. Then we scraped among the cells with 200 μl pipette tips followed by added the serum-free medium. Wound closure was photographed with an Olympus IX 71 inverted fluorescence microscope (Olympus, Tokyo, Japan) at 0 and 24 h after scratch. Diminishing area among scratch wound was analyzed, normalized to the control group (0 h group).

**Transwell migration and Matrigel invasion assays**

We coated Transwell chamber (Corning Costar, Corning, NY, USA) with or without Matrigel (BD Science, Bedford, MA, USA) for either invasion or migration verification. Briefly, medium supplemented with 20% FBS was added to the lower chambers as chemoattractant, and 5 × 10^4^ cells for migration or 1 × 10^5^ OS cells for invasion verification were suspended in serum-free medium and added to the upper chambers. After co-culture for 24 h, cells in the filter were fixed followed by 0.1% crystal violet staining, the migrated or invaded cells were photographed and counted using an Olympus IX 71 inverted fluorescence microscope (Olympus, Tokyo, Japan). Quantity statistics and quantified were conducted in three random visual fields. For image quantification, at least 5 randomly selected images were chosen from each independent experiment, and the mean level of 3 independent experiment were quantified at last.

**Magnetic endothelial cells separation**

Dynabeads conjugated to the anti-CD31 antibody (ab28364, Abcam) were prepared using the Dynabeads Antibody Coupling Kit (Life Technologies, 14311D) according to the manufacturer’s instruction. Fresh osteosarcoma tissue excised from patients with or with metastasis were washed multiple times with 1X PBS buffer and gentle agitation to wash away the bone fragments (approximately three to six times, or until the buffer is no longer red/pink). The tumor tissues were minced with a scalpel into 1–2mm pieces in the presence of 0.25% collagenase I (Yeasen, Shanghai, China) solution (in PBS + 20% FBS), and then digested in the 0.25% collagenase solution for 45 min in a 37°C shaking incubator. After digestion, cells were filtrated through sterile 70μm nylon mesh and washed with cold 1X PBS buffer. Conjugated dynabeads were added to cells and incubated for 30 min at 4°C to allow binding to cells. The bead-bound cells were washed four times in 1X PBS buffer and collected using a magnet. All bead-bound cells were resuspended in DMEM with 20% FBS prior to plating.

**Tube formation assay**

We performed the tube formation assay according to the published literature[5]. Briefly, the Matrigel (BD Sciences, Bedford, MA, USA) were coated on 96-well culture plates and stilled standing at 37 ℃ for 30 min until gelation. Then, we seeded HUVEC cells (15 × 10^3^ cells per well) into culture plates and incubated for 6 h followed by Calcein-AM (Yeasen, Shanghai, China) staining. Green fluorescence was photographed with an Olympus IX 71 inverted fluorescence microscope (Olympus, Tokyo, Japan) and total tube formation was analyzed by ImageJ plug-in for angiogenesis (NIH, <http://rsb.info.nih.gov/ij/>). For image quantification, at least 3 randomly selected images were chosen from each independent experiment, and the mean level of 3 independent experiment were quantified at last.

**Aortic ring assay**

We conducted aortic ring assay referring to previously report[6]. In brief, thoracic aortas were excised from mice (C57BL/6 mice, male, 8-10 weeks old) and cut into wing approximately 1 mm in width. The rings were then embedded per well of a 96-well culture plates containing 50μL of collagen type I (Millipore, Billerica, MA, USA) and transfection were performed under sterile conditions. After incubation for 1 week, the aortic rings were fixed with paraformaldehyde and specifically stained with fluorescent antibody, finally photographed using a Zeiss LSM 880 confocal microscopy (Carl Zeiss, Oberkochen, Germany). The microvessel area was quantified by TRI2 (<http://www.assembla.com/spaces/ATD_TRI/wiki>).

**Chick embryo chorioallantoic membrane (CAM) assay**

We performed CAM assay based on published literature[7]. We bought fertilized sterile eggs from Boehringer Ingelheim (Beijing, China) and cut a square window into shell after 48 h incubation. After sealing the windows, eggs were incubated at 37 ℃ for another 6 days. Until 8 day of incubation, we opened the windows and placed sterilized gelatin sponge containing transfection reagents onto the CAMs, assays for each group using at least 6 eggs. The eggs were incubated until day 12, the blood vessel areas on CAM were quantitatively analyzed according to published literature[8] using the ImageJ software. The branching points were analyzed by ImageJ plug-in for angiogenesis.

**Dual-luciferase reporter assay**

Reporter plasmids (Hanbio, Shanghai, China) was co-transfected with either miRNA mimics, YY1 vector or negative control (RiboBio, Guangzhou, China) in HEK-293 T cells. After 48 h incubation, firefly luciferase activity measured by Luciferase Reporter Gene Assay Kit (Beyotime, Shanghai, China) was normalized to Renilla luciferase activity for comparisons.

**RNA immunoprecipitation (RIP)**

The Ago-RIP was conducted using RNA Immunoprecipitation Kit (Geneseed, Guangzhou, China) as previously described[9]. 1 ml RIP lysis Buffer containing protease inhibitors cocktail and RNase inhibitors RIP was added to cell pellet after centrifugation. The supernatant of lysate was divided into two parts and respectively incubated with Ago2 antibody- (proteintech, USA) and IgG-magnetic bead complexes followed by uniform rotation at 4 °C for 2 h. Total RNAs were purified and extracted from bead complexes, the mRNA and circFIRRE components levels were then measured by RT-qPCR.

**Pull-down assay**

Biotinylated circFIRRE, FIRRE and miRNAs and control probes were designed and synthesized by RiboBio (Guangzhou, China). 1 × 10^7^ cells were harvested and lysed by pull-down lysis Buffer. Biotinylated probes were incubated with C-1 magnetic beads (Life Technologies) to generate probe-beads complexes at 25 °C for 2 h, then probe-beads complexes were incubated with the pre-prepared supernatant of lysates to pull-down at 4 ℃ overnight. Total RNAs were eluted and extracted from probe-beads complexes for RT-qPCR.

**RNA Fluorescence in situ hybridization (FISH)**

Cy3-labeled circFIRRE probes, FAM-labeled miRNAs probes and FISH Kit were provided by RiboBio (Guangzhou, China). All operations were conducted referring to the manufacturer’s instructions. The probe signals were detected using a Zeiss LSM 880 confocal microscopy (Carl Zeiss, Oberkochen, Germany) and analyzed by ImageJ.

**Immunohistochemistry (IHC)**

The paraformaldehyde-fixed and paraffin-embedded tissues were immunostained with anti-LUZP1 antibody (1:500, proteintech, USA), anti-Ki-67 (1:50, BD Biosciences, USA), anti-E-Cadherin (1:400, Cell Signaling Technology, USA), anti-N-cadherin (1:1000, proteintech, USA), anti-Vimentin (1:2500, proteintech, USA), anti-CD31 (1:300, Servicebio, Wuhan, China), anti-VEGF (1:100, Santa Cruz Biotechnology, USA) at 4 ℃ for one night. Horseradish peroxidase (HRP)-labeled goat anti-mouse and anti-rabbit secondary antibodies (Zsgb-bio, Beijing, China) were used as appropriate. After incubation using a DAB Substrate Kit (Abcam, USA), the staining was visualized. Finally, we assessed the relative expression level via percentage of positive areas.

**In vivo experiments**

BALB/c Nude mice (male, 4 weeks old) were bred and provided from GemPharmatech (Jiangsu, China). All experiments involving animals were conducted comply with the Guide for the Care and Use of Laboratory Animals published by National Institutes of Health and approved by the Animal Ethical and Welfare Committee of Nanjing University (IACUC-2101002). All experiments strictly observed the panel’s specific guidelines in regard to the care, treatment and euthanasia of animals used in this study.

In orthotopic xenograft tumor models to verify the effect of circRNA-miRNAs axis on OS, 5 × 10^6^ MG63 cells labeled with luminescent dye were suspended in sterile PBS followed by tibial bone marrow cavity injection (10 mice in each group) using a microliter syringe (Hamilton, Bonaduz, Switzerland). 4 weeks after injection, we intraperitoneal injected 150 mg/kg D-Luciferin firefly, sodium salt monohydrate (Yeasen, Shanghai, China) into mice and observed the tumor progression via bioluminescent in a IVIS Spectrum In Vivo Imaging System (PerkinElmer, USA), tumor induced-bone destruction was evaluated using a Hiscan XM Micro-CT (Hiscan, Suzhou, China) 5 weeks after injection.

In tail vein metastasis models to verify the effect of circRNA-miRNAs axis on OS, a total of 5 × 10^6^ luminescence-labeled MG63 cells were suspended and injected into the mouse tail vein (10 mice in each group). 4 weeks after injection, lung metastasis was assessed by IVIS Spectrum In Vivo Imaging System (PerkinElmer, USA), and specific location and size of metastatic tumors were analyzed by micro-CT (Hiscan, Suzhou, China).

**References**

1. Ang YS, Rivas RN, Ribeiro AJS, Srivas R, Rivera J, Stone NR, Pratt K, Mohamed TMA, Fu JD, Spencer CI, et al: **Disease Model of GATA4 Mutation Reveals Transcription Factor Cooperativity in Human Cardiogenesis.** *Cell* 2016, **167:**1734-1749 e1722.

2. Yu G, Wang LG, Han Y, He QY: **clusterProfiler: an R package for comparing biological themes among gene clusters.** *Omics* 2012, **16:**284-287.

3. Sanger F, Nicklen S, Coulson AR: **DNA sequencing with chain-terminating inhibitors.** *Proc Natl Acad Sci U S A* 1977, **74:**5463-5467.

4. Wu N, Yuan Z, Du KY, Fang L, Lyu J, Zhang C, He A, Eshaghi E, Zeng K, Ma J, et al: **Translation of yes-associated protein (YAP) was antagonized by its circular RNA via suppressing the assembly of the translation initiation machinery.** *Cell death and differentiation* 2019, **26:**2758-2773.

5. Arnaoutova I, Kleinman H: **In vitro angiogenesis: endothelial cell tube formation on gelled basement membrane extract.** *Nature protocols* 2010, **5:**628-635.

6. Baker M, Robinson S, Lechertier T, Barber P, Tavora B, D'Amico G, Jones D, Vojnovic B, Hodivala-Dilke K: **Use of the mouse aortic ring assay to study angiogenesis.** *Nature protocols* 2011, **7:**89-104.

7. Ribatti D, Nico B, Vacca A, Presta M: **The gelatin sponge-chorioallantoic membrane assay.** *Nature protocols* 2006, **1:**85-91.

8. Magnaudeix A, Usseglio J, Lasgorceix M, Lalloue F, Damia C, Brie J, Pascaud-Mathieu P, Champion E: **Quantitative analysis of vascular colonisation and angio-conduction in porous silicon-substituted hydroxyapatite with various pore shapes in a chick chorioallantoic membrane (CAM) model.** *Acta Biomater* 2016, **38:**179-189.

9. Gagliardi M, Matarazzo MR: **RIP: RNA Immunoprecipitation.** *Methods Mol Biol* 2016, **1480:**73-86.

**Primers, probes, siRNAs and shRNAs used in this study**

| **List of oligonucleotides** | **Sequences （5‘-3’）** |
| --- | --- |
| **primers for RT-PCR and RT-qPCR** | |
| circFIRRE-F | GAGAGGAGATACTTTATGAGGAGACTAAGG |
| circFIRRE-R | GCAAGCCAGGTACAGTCTTGTG |
| FIRRE-F | TGTCTGTGTAACTGAGGTGCT |
| FIRRE-R | GCTTAGAAAAGGATTGTGGCAGA |
| YY1-F | GGATAACTCGGCCATGAGAA |
| YY1-R | ATAGGGCCTGTCTCCGGTAT |
| LUZP1-F | GGAATCGGGTAGGAGACACCA |
| LUZP1-R | TTCCCAGGCAGTTCAGACGGA |
| GAPDH-F | GGAGCGAGATCCCTCCAAAAT |
| GAPDH-R | GGCTGTTGTCATACTTCTCATGG |
| hsa_circ_0003563-F | GGAACCCAGAAGTGGTAGCC |
| hsa_circ_0003563-R | CATCGTTACCCGCCATGACA |
| hsa_circ_0003915-F | GCCCTCTCTCCCAGAGTATG |
| hsa_circ_0003915-R | CTGCTCCACGACACAAAAGAC |
| hsa_circ_0001947-F | ACACTCTTGGATGGAAAACCCA |
| hsa_circ_0001947-R | CGTGTTCTGGACTCGGTTGG |
| hsa_circ_0003162-F | CTCAGGAACCTTGGGTAATGTG |
| hsa_circ_0003162-R | CCACTATTGTCAACATTAGCCAGA |
|  | |
| **RNA pull-down probes** | |
| circFIRRE-1 | CCTTAGTCTCCTCATAAAGT(3biotin) |
| circFIRRE-2 | GACACCTTAGTCTCCTCATA(3biotin) |
| FIRRE | GACCACGCACAAACAGAUGAGAACCAAAACCGAGUGAA(3biotin) |
| control | ACTCTACGCTCTCACCATCC(3biotin) |
| miR-486-3p | （5biotin)CGGGGCAGCUCAGUACAGGAU |
| miR-1225-5p | （5biotin)GUGGGUACGGCCCAGUGGGGGG |
|  | |
| **siRNAs** | |
| circFIRRE siRNA-1 | GAGAUACUUUAUGAGGAGACU |
| circFIRRE siRNA-2 | ACUUUAUGAGGAGACUAAGGU |
| circFIRRE siRNA-3 | UAUGAGGAGACUAAGGUGUCA |
| YY1 siRNA-1 | CGACGACTACATTGAACAA |
| YY1 siRNA-2 | CGGCTTCGAGGATCAGATT |
| YY1 siRNA-3 | CACCATGTGGTCCTCAGAT |
| FIRRE siRNA-1 | CCAUGUACACCAUCAUCAA |
| FIRRE siRNA-2 | GCCUAGGACCUUUGUGGUA |
| LUZP1 siRNA | GAAGACAACACGAACGUUU |
|  | |
| **shRNAs** | |
| circFIRRE shRNA-1 F | ccggGAGATACTTTATGAGGAGACT**CTCGAG**AGTCTCCTCATAAAGTATCTCtttttg |
| circFIRRE shRNA-1 R | aattcaaaaaGAGATACTTTATGAGGAGACT**CTCGAG**AGTCTCCTCATAAAGTATCTC |
| circFIRRE shRNA-2 F | ccggACTTTATGAGGAGACTAAGGT**CTCGAG**ACCTTAGTCTCCTCATAAAGttttttg |
| circFIRRE shRNA-2 R | aattcaaaaaACTTTATGAGGAGACTAAGGT**CTCGAG**AGTCTCCTCATAAAGTATCTC |
