# Supplementary material for: Circular RNA circFIRRE drives osteosarcoma progression and metastasis through tumorigenic-angiogenic coupling
Source: Mol Cancer. 2022 Aug 19;21:167. doi: 10.1186/s12943-022-01624-7 (PMC9389772; doi:10.1186/s12943-022-01624-7)

A

Characteristic	circFIRRE High	circFIRRE Low	P value
All cases	52	52	
Gender, n (%)			0.025
Female	13 (12.5%)	25 (24%)	
Male	39 (37.5%)	27 (26%)	
Age, n (%)			< 0.001
<16	18 (17.3%)	42 (40.4%)	
≥16	34 (32.7%)	10 (9.6%)	
Metastatic, n (%)			< 0.001
metastatic	30 (28.8%)	10 (9.6%)	
non-metastatic	22 (21.2%)	42 (40.4%)	
Histologic response, n (%)			0.752
Stage 1/2 (0-90 % necrosis)	25 (30.1%)	27 (32.5%)	
Stage 3/4 (91-100 % necrosis)	13 (15.7%)	18 (21.7%)	
Primary tumor site, n (%)			0.504
Arm/hand	6 (5.8%)	5 (4.9%)	
Leg/foot	43 (41.7%)	41 (39.8%)	
Other	0 (0%)	2 (1.9%)	
Pelvis	3 (2.9%)	2 (1.9%)	
Skull	0 (0%)	1 (1%)	
Definitive Surgery, n (%)			0.525
Amputation	5 (5.1%)	8 (8.2%)	
Limb sparing	45 (45.9%)	37 (37.8%)	
No surgery	2 (2%)	1 (1%)	
Vital Status, n (%)			< 0.001
Alive	15 (14.4%)	39 (37.5%)	
Dead	36 (34.6%)	13 (12.5%)	
Unknown	1 (1%)	0 (0%)	

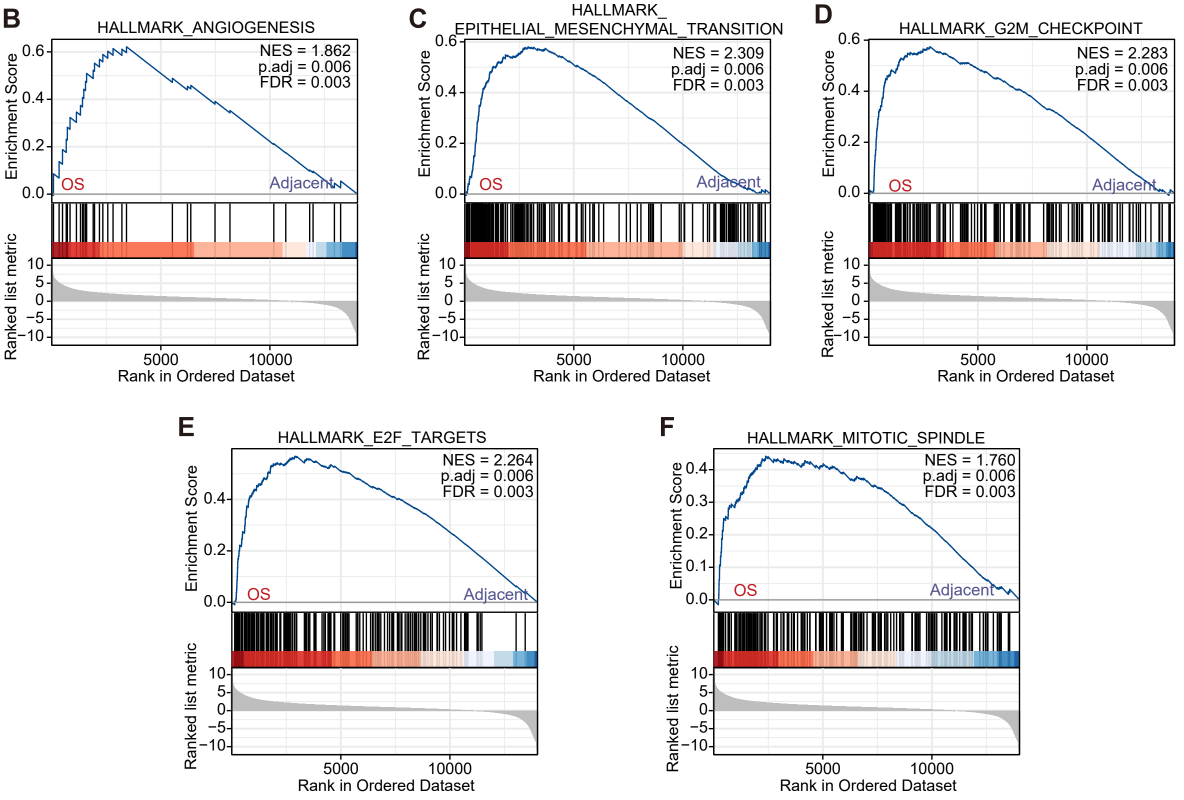

Supplement: Supplementary file 2 — Additional file 2: Figure S1. Validation of differentially expressed circRNAs in RNA-seq. (A) The length distribution of aberrantly expressed circRNAs in RNA-seq. (B) 30 upregulated circRNAs were screened out through filter criteria as described and sorted by name. (C) Quantification of the fluorescence intensity of circFIRRE in FISH assay (n=5 in each group). (D) Online prediction algorithms (lncLocator, www.csbio.sjtu.edu.cn/bioinf/lncLocator) was applied to explore the intracellular localization of circFIRRE in OS cells. (E) Quantification of the fluorescence intensity of circFIRRE in both nucleus and cytoplasm in FISH assay (n=15 in each group). Figure S2. Baseline clinical data and GSEA analysis data. (A) Clinical baseline characteristics of 104 OS patients. (B-F) GSEA (https://www.gsea-msigdb.org/gsea/index.jsp) analysis of hallmark gene sets showed that highly expressed circFIRRE was associated with epithelial mesenchymal transition (EMT), cell cycle (E2F targets, G2M checkpoint and mitotic spindle) and angiogenesis. Figure S3. circFIRRE knockdown can inhibit OS progression in vitro. (A) Three circFIRRE-specific shRNAs were designed and the knockdown efficiency were verified by RT-qPCR in both MG63 and U2OS (n=3 in each group). (B) CCK8 assay was applied to estimate cell viability influenced after transient transfection of siRNAs (si-circFIRRE-1 and -2) at different time points in both MG63 and U2OS (n=6 at each time point). (C) Wound healing assay exhibited cell migration. Scar bar=200 μm. (D) Schematic of the lentiviral vector GV344 (hU6-MCS-Ubiquitin-firefly_Luciferase-IRES-puromycin). (E) Wound healing assay exhibited cell migration after stable infection of lentivirus in MG63 and U2OS cells. Scar bar=200 μm. (F-H) Flow cytometry analysis of cell cycle distribution. (G-H) Quantification of cell cycle in MG63 and U2OS cells (n=3 in each group). Values are presented as mean ± SD; the bar charts, line charts, error bars and dots represent the quantitative an [file 12943_2022_1624_MOESM2_ESM.zip › figure S2_300ppi_ESM.pdf]
